# Multi-sensor monitoring of a transient event in the Gran Sasso aquifer, Italy

**DOI:** 10.1038/s41598-025-33923-6

**Published:** 2026-02-11

**Authors:** Marino Domenico Barberio, Andrea Basti, Thomas Braun, Giorgio Carelli, Simone Castellano, Gaetano De Luca, Giuseppe Di Carlo, Giuseppe Di Somma, Angela D. V. Di Virgilio, Daniela Famiani, Francesco Fuso, Francesca Gori, Aladino Govoni, Valeria Lorenzi, Enrico Maccioni, Paolo Marsili, Marco Petitta, Luca Pizzino, Ezio Previtali, Marco Tallini

**Affiliations:** 1 Istituto Nazionale di Geofisica e Vulcanologia (INGV) - Sez. Roma1 Sez. Roma1, Roma, Italy; 2 Dipartimento di Fisica, Università di Pisa, Pisa, Italy; 3 Istituto Nazionale di Fisica Nucleare (INFN) - Sez. Pisa, Pisa, Italy; 4 Istituto Nazionale di Geofisica e Vulcanologia (INGV) - Sez. Roma1, Arezzo, Italy; 5 Istituto Nazionale di Geofisica e Vulcanologia (INGV), Sez. Osservatorio Nazionale Terremoti (ONT), L’Aquila, Italy; 6 Istituto Nazionale di Fisica Nucleare (INFN), Laboratori Nazionali del Gran Sasso (LNGS), L’Aquila, Italy; 7 Università “La Sapienza” - Dipartimento Scienze della Terra, Roma, Italy; 8 Istituto Nazionale di Geofisica e Vulcanologia (INGV), Sez. Osservatorio Nazionale Terremoti (ONT), Roma, Italy; 9 Dipartimento di Ingegneria Civile, Edile - Architettura e Ambientale, Università degli Studi dell’Aquila, L’Aquila, Italy

**Keywords:** Environmental sciences, Hydrology, Natural hazards, Solid Earth sciences

## Abstract

A novel approach in monitoring the inner dynamics of mountains, massifs, and of the Earth crust in general, involves hydrogeological measurements, as well as new generation multi-component seismic stations. Several hydrogeological stations monitor the water parameters of the large Gran Sasso aquifer. This is especially important when the instrumentation used has a high sensitivity and is able to access frequencies below 1 mHz, opening the possibility of observing very slow signals of local origin. For several years, the ring laser gyroscope *GINGERINO* is operative inside the underground Gran Sasso laboratory (*LNGS-INFN*), and monitors the local Earth angular velocity around the vertical axis. Together with the co-located *GIGS* broadband seismometer (seismic stations of national network of *INGV*), it constitutes a *4C* seismic station; the 4 degrees of freedom put together give insight into the inner movements of the Gran Sasso massif, that find correspondence in measurements conducted on the groundwater of the aquifer. In particular, the hydrogeological interpretation of the slow dynamics of the period since May to August 2023, and of the powerful mountain *bang* event of August 14 is consistent with data from *GINGERINO*. The final large *bang* event was also detected by the *GIGS* broadband seismic station and accelerometer station of *RAN* (National Accelerometer Network of Civil Protection Department). Furthermore, in the underground laboratories the *bang* event was recorded by an acoustic sensor and the groundwater hydraulic pressure monitor shows an anomaly exactly when the *bang* event occurred. Finally, the monitoring data of the groundwater at the boundary of the Gran Sasso aquifer also reveal anomalies linked to the *bang* event.

## Introduction

Acoustic phenomena during earthquake ruptures have been described manifold, as, e.g., for the great 1964 Alaska earthquake^1^. Natural sources, such as shallow earthquakes can generate infrasound,an overview can be found in^2–4^. Earthquake infrasound is caused by low-frequency vertical ground motion near the epicenter and elsewhere that creates atmospheric pressure fluctuations^3–6^, sometimes producing audible sounds in the frequency range ~20 to 70 Hz^5^, as a consequence of a decoupling of the P-wave into the atmosphere.

In a volcanic environment, sound generation is hypothesized to result from multi-phase fluid flow within the volcanic edifice (see ^7^ for an overview),this process can be observed even in absence of eruptive activity. Martinelli^8^ modelled the two-phase system of magma as a homogeneous but a compressible fluid. For shallow depths, the gas phase in the liquid greatly reduces the propagation velocity of the speed of sound^9^. At a velocity of the order of a few meters per second (Mach M = 1), and under particular geometrical conditions, such as a conduit which undergoes sudden enlargement of its cross-section, trans-sonic flow (i.e., mixed regions where Mach < 1 and Mach > 1) occurs. Under certain pressure regimes, the flow undergoes periodic density and velocity oscillations. The oscillations generate pressure fluctuations which are transmitted to the confining rock structure audible sound and are larger than those cast transmitted by mixing turbulence.

**Fig. 1:**
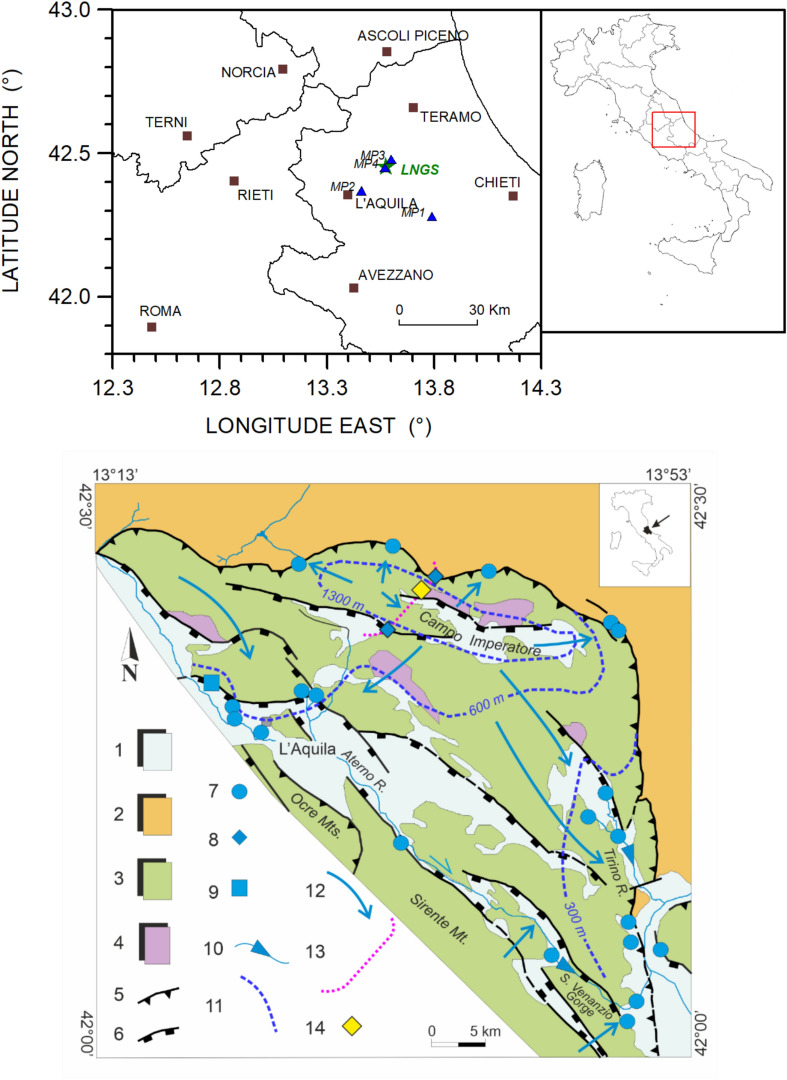
Top: Location map of underground Gran Sasso laboratory (*LNGS-INFN*) with a green star; brown squares represent principal towns in the area, the black lines are for regional administrative boundaries and blue triangles are the hydrogeological monitoring stations (*MP1*: Tirino springs area of South-East Gran Sasso aquifer, *MP2*: Tempera spring area of South-West Gran Sasso aquifer, *MP3*: the northern drainage area of Gran Sasso motorway tunnel, *MP4*: hydraulic pressure sensor inside the motorway tunnel near *LNGS-INFN*). Bottom: Gran Sasso simplified hydrogeological map. 1 – Quaternary detrital deposits (aquitard); 2 – Neogene terrigenous deposits (aquiclude); 3 – Meso-Cenozoic carbonate rocks (aquifer); 4 – Upper Triassic dolomite (aquiclude); 5 – overthrust (permeability boundary); 6 – extensional fault; 7 – main spring; 8 – highway tunnels drainage; 9 – well field for drinkable purpose; 10 – linear spring; 11 – water table contour line; 12 – groundwater flowpath; 13 – Gran Sasso highway tunnels; 14 – *LNGS-INFN* (modified from Tallini et al.^14^).

On August 14, 2023 (day of the year, DOI: 226) at 22:00 UTC, a large acoustic *bang* event was heard in the underground Gran Sasso laboratory (*LNGS-INFN*) by the personnel present on the night shift. Figure 1 shows both a simplified hydrogeological map of the area and a location map of the Gran Sasso underground laboratory. This type of phenomenon is not new to the scientific community (see Hill^10^ and reference inside). It is often associated with the occurrence of shallow earthquakes, the triggering of landslides, and turbulent motions in highly fractured and karstified aquifers^11–13^. In hydrogeology, such reports often correlate with the breach of local permeability boundaries, cavitation processes, or turbulent flows within fractured and karstified aquifers. In addition to the testimonies of the operators working inside the *LNGS*-*INFN* laboratories, the simultaneous presence of several experiments from various disciplines (physics, seismology, and hydrogeology), all investigating the Gran Sasso massif and beyond, allowed for the collection of an enormous amount of data before, during, and after the occurrence of the cited acoustic *bang* event.

Hydrogeological data were compared with seismological data, including rotational seismology data provided by the *GINGERINO* Ring Laser Gyroscope (*RLG*) located underground at *LNGS-INFN*. The *RLG* is co-located with the *GIGS* seismic station, which is equipped with a 3-component broadband sensor (Nanometrics, Trillium 240 s). *GINGERINO*, which is a prototype of the *GINGER* experiment in construction in 2026-27, shows an unprecedented sensitivity to the aquifer movements, not detected by the *GIGS* broadband seismometer, providing insight observations of the mechanisms regulating the aquifer changes due to water-rock physical interactions.

## Hydrogeological setting of gran sasso aquifer (central apennine)

The Gran Sasso range is mostly made of Mesozoic to Cenozoic limestones stacked and translated towards the Laga Basin^15^. The frontal structure consists of an upper and a lower thrust^16,17^. The entire carbonate sequence was subsequently affected by an extensional tectonic phase. This tectonic activity lowered entire sectors of the massif (e.g., the *Campo Imperatore* basin), partly through the formation of new extensional faults (such as the Assergi-Valle Fredda, the *Campo Imperatore*, and the Tre Selle faults) and partly through the extensional reactivation of older thrust faults^18^. The boundary between carbonate rocks and, to the north and east, the Upper Miocene-Lower Pliocene siliciclastic lithologies and, to the south, the Plio-Quaternary detrital deposits, acts as clear no-flow boundary for the former and partially one for the latter^19^. Nowadays, the Gran Sasso massif, with an area of about 1000 km^2^, represents one of the largest karst-fractured carbonate aquifers in central-southern Italy^20^.

**Fig. 2:**
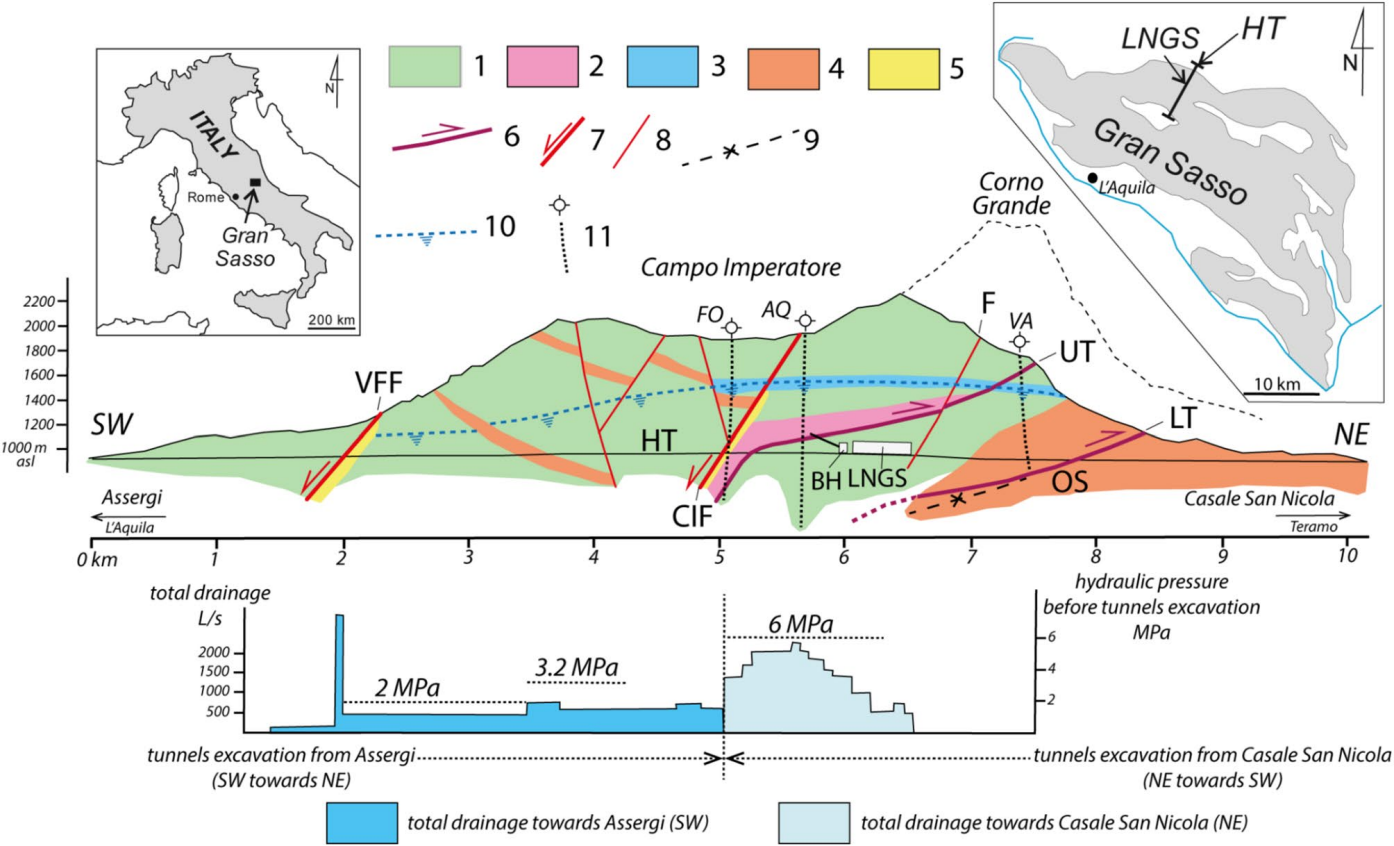
Top: Gran Sasso hydrogeological section along the highway tunnels (HT) and the *LNGS-INFN* and the borehole hall (BH). **1-** limestone (Upper Cretaceous - Upper Jurassic); **2-** dolomite (Upper Triassic); **3-** paleokarst horizon; **4-** siliciclastic low-permeability lithologies; **5-** low-permeability fault rock; **6-** main thrust (UT: upper thrust; LT: lower thrust); **7-** main normal fault (CIF: *Campo Imperatore* fault; VFF: Valle Fredda fault); **8-** minor normal fault (F behaves hydraulically as a drain); **9-** Overturned syncline (OS) in the UT footwall; **10-** water table before the tunnels excavation; **11-** deep boreholes (FO: Fontari; AQ: Monte Aquila; VA: Vaduccio). Bottom: groundwater drainage during the excavation work (modified from Isaya et al.^31^).

**Fig. 3:**
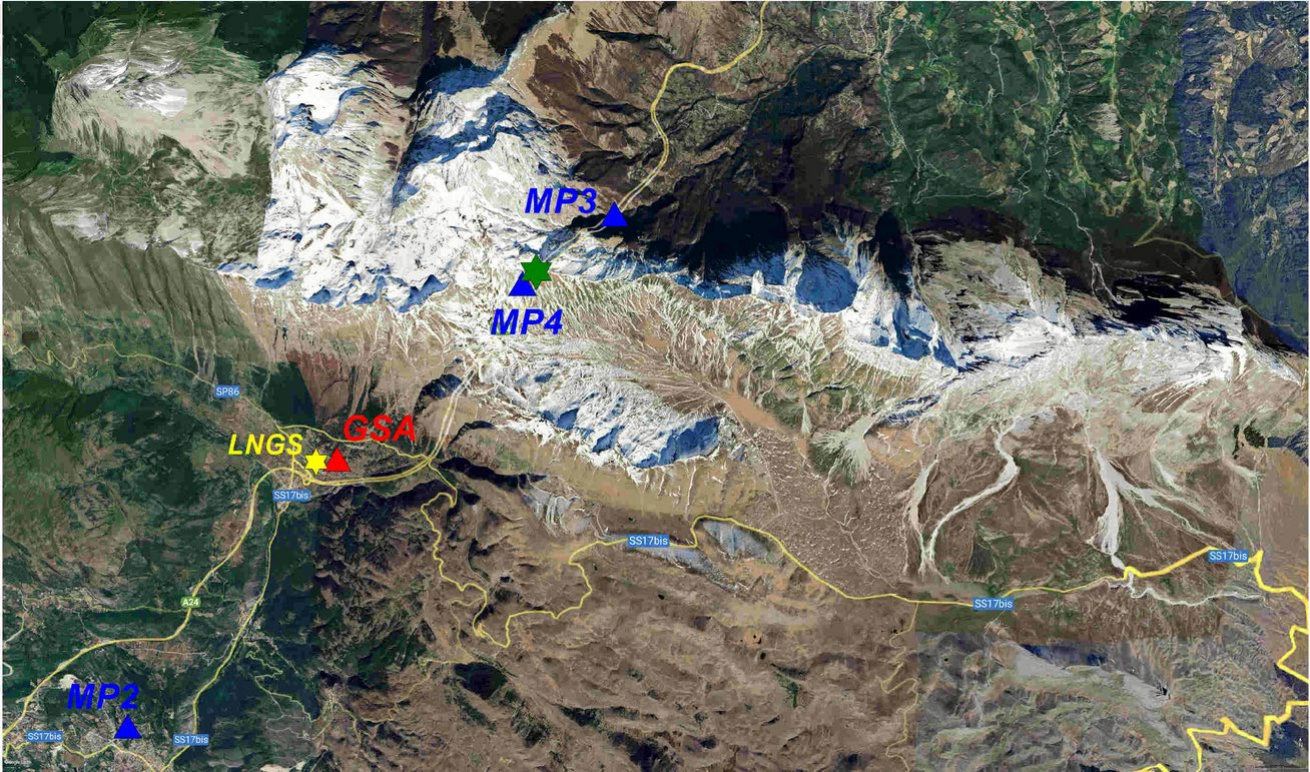
Map (image source: *Google Earth*, retrieved from https://earth.google.com) of the monitoring points external to the underground laboratories site (green star); blue triangles are the hydrogeological monitoring stations: Tempera spring area of South-West Gran Sasso aquifer (*MP2)*, the northern drainage area of Gran Sasso motorway tunnel (*MP3*), and hydraulic pressure sensor inside the motorway tunnel near *LNGS-INFN* (*MP4*); yellow star represent the position of external office of *LNGS-INFN* and the red triangle is the position of *GSA* accelerometer station of *RAN* (https://ran.protezionecivile.it/IT/quakelive.php).

**Fig. 4:**
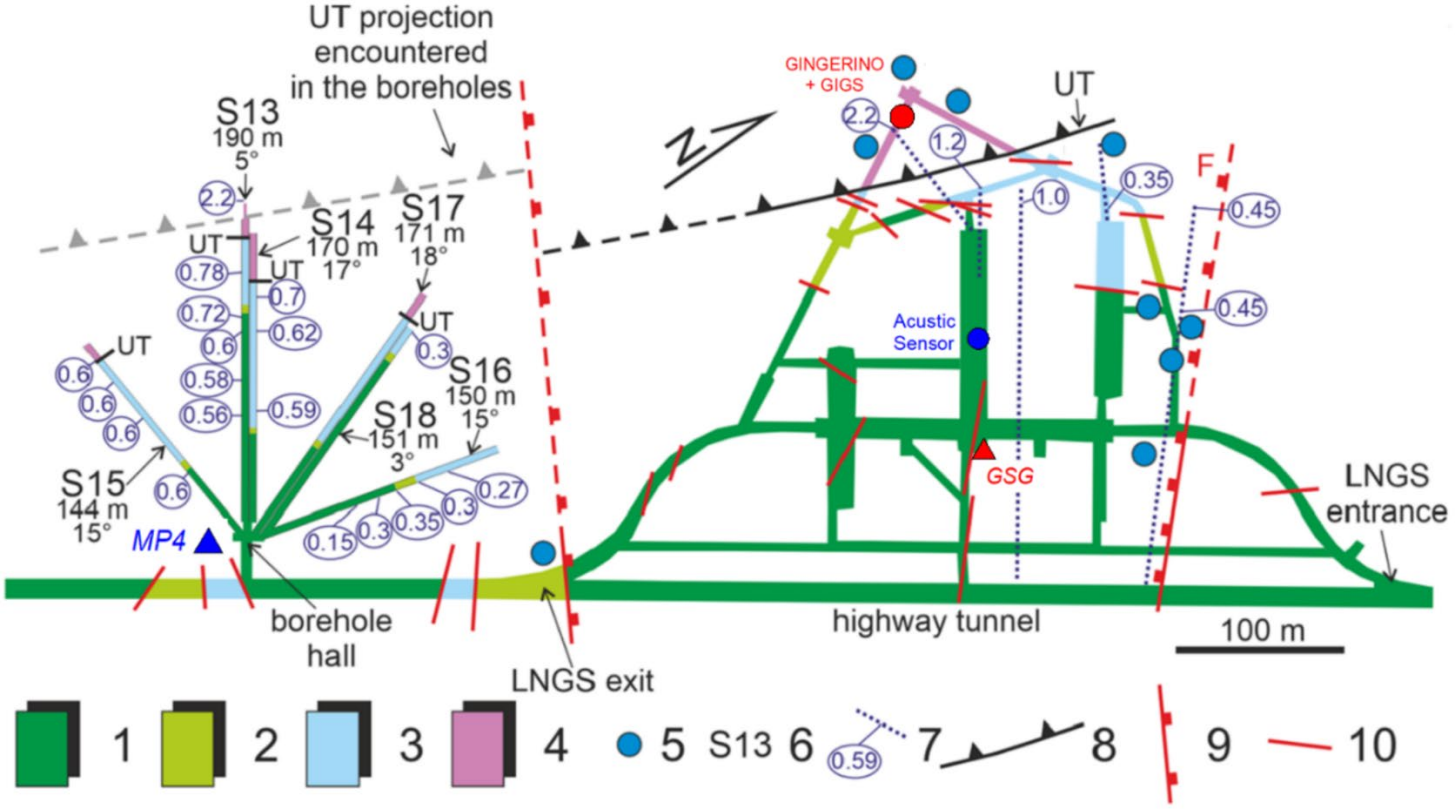
*LNGS-INFN* hydrogeological map at right side with the three main parallel experimental halls, and borehole hall at left side. **1 –** stratified limestone (Upper Cretaceous); **2 –** detrital massive limestone with local marly intercalations (middle Cretaceous); **3 –** stratified limestone with cherty layers (Lower Cretaceous - Upper Jurassic); **4 –** stratified dolomite (Upper Triassic); the dolomite close to the thrust UT, belonging to its fault damage zone, is strongly tectonized; **5 –** remarkable concentrated groundwater flow; **6 –** borehole with whole length (m) and dip angle (°); **7 –** hydraulic pressure (MPa) measured during borehole drilling; **8 –** upper thrust (UT), dashed if inferred; **9 –** minor normal fault, dashed if inferred; **10** – fault. In addition: red circle indicates the location of *GINGERINO* and *GIGS*, blue circle the microphone, red triangle the accelerometer station of *RAN*, and the blue one indicates the hydraulic pressure measurements at *MP4* (redrawn from Isaya et al.^31^).

This fractured and partially karstified aquifer, whose basal flow can be considered a single on a regional scale, feeds many significant springs located at its boundaries. Springs are characterized by a total discharge ranging from 18 and 25 m^3^/s, corresponding to a recently recalculated net infiltration of about 600 mm/y^21^. The preferential recharge zone has been identified in the endorheic basin of *Campo Imperatore*^14,22^, located at the ridge core. Its tectonic-glacial-karstic origin enhances the infiltration and the sub-vertical flow of infiltrating waters^23^. Once reaching the saturated zone, the groundwater flows partly along tectonic discontinuities, which act as preferential flow path and partly along the natural hydraulic gradient until reaching the springs located at the aquifer boundaries. The construction of the Gran Sasso motorway tunnel (two 10.2 km long tunnels, each for one direction), completed in 1980, certainly interfered with the groundwater circulation^24–^^28^. Figure 2 shows the hydrogeological cross-section of the Gran Sasso massif obtained during the excavation work of the motorway tunnels^29,30^. Groundwater pressures reaching more than 6 MPa were measured during the excavation, which corresponds to approximately 600 m of water pressure head,currently, the maximum pressures recorded are around three MPa (De Luca et al., 2018). After stabilization, the aquifer reached a new hydrodynamic equilibrium with flows of approximately 2.0 m^3^/s towards the north exit, and 0.6 m^3^/s towards the south exit of the tunnels. Figures 3 and 4 show the hydrogeological underground map of *LNGS-INFN* with the location of the *GINGERINO* experiment and co-located *GIGS* broadband seismometer of National Seismic Network of *INGV* (https://terremoti.ingv.it/en/instruments/station/GIGS),the locations for the *MP4* and microphone measurements are also indicated.

From a hydrogeological perspective, this work focused on the results obtained from the hydrogeological monitoring carried out in four discharge areas of the Gran Sasso regional aquifer. Specifically: *(a)* the area of the Tirino springs group (*MP1*), *(b)* the Tempera Spring (*MP2*), *(c)* the northern drainage of the motorway tunnel (*MP3*), and *(d)* the hydraulic pressures recorded inside the motorway tunnel (*MP4*); see Figures 1, 2, 3 and 4.

## Data presentation

### Hydrogeological monitoring

The hydrogeological data from four monitoring stations, based on the spring characteristics, were assessed^32^. Two sites were selected in the main discharge zone of the southern side of Gran Sasso aquifer (*MP1* and *MP2*), and two sites related to the drainage inside and outside the Gran Sasso motorway tunnel (*MP3* and *MP4*), see Figures 1, 3 and 4. At the stations *MP1*, *MP2*, and *MP3,* hydrogeological parameters were measured using the *OTT ecoLog 800* multi-parametric probe, equipped by an automatic system for continuous acquisition and remote data transmission, including groundwater level, temperature, and electrical conductivity (groundwater level: resolution 0.001 m, error ±0.05%,temperature: resolution 0.001 °C, error 0.1 °C,electrical conductivity: resolution 0.001 mS/cm, error ±0.5%). The frequency of data measurements was 30 minutes. The groundwater level measurements were automatically adjusted to account for variations in barometric pressure. A different monitoring system was installed at station *MP4* (there are six horizontal drainage boreholes of about 200 m of length, see Figure 4): a 3-channel, 24-bit ADC data logger (mod. *SL06* by *Sara Electronic Instruments* – http://www.sara.pg.it/), configured for continuous local recording since 2015.

**Fig. 5:**
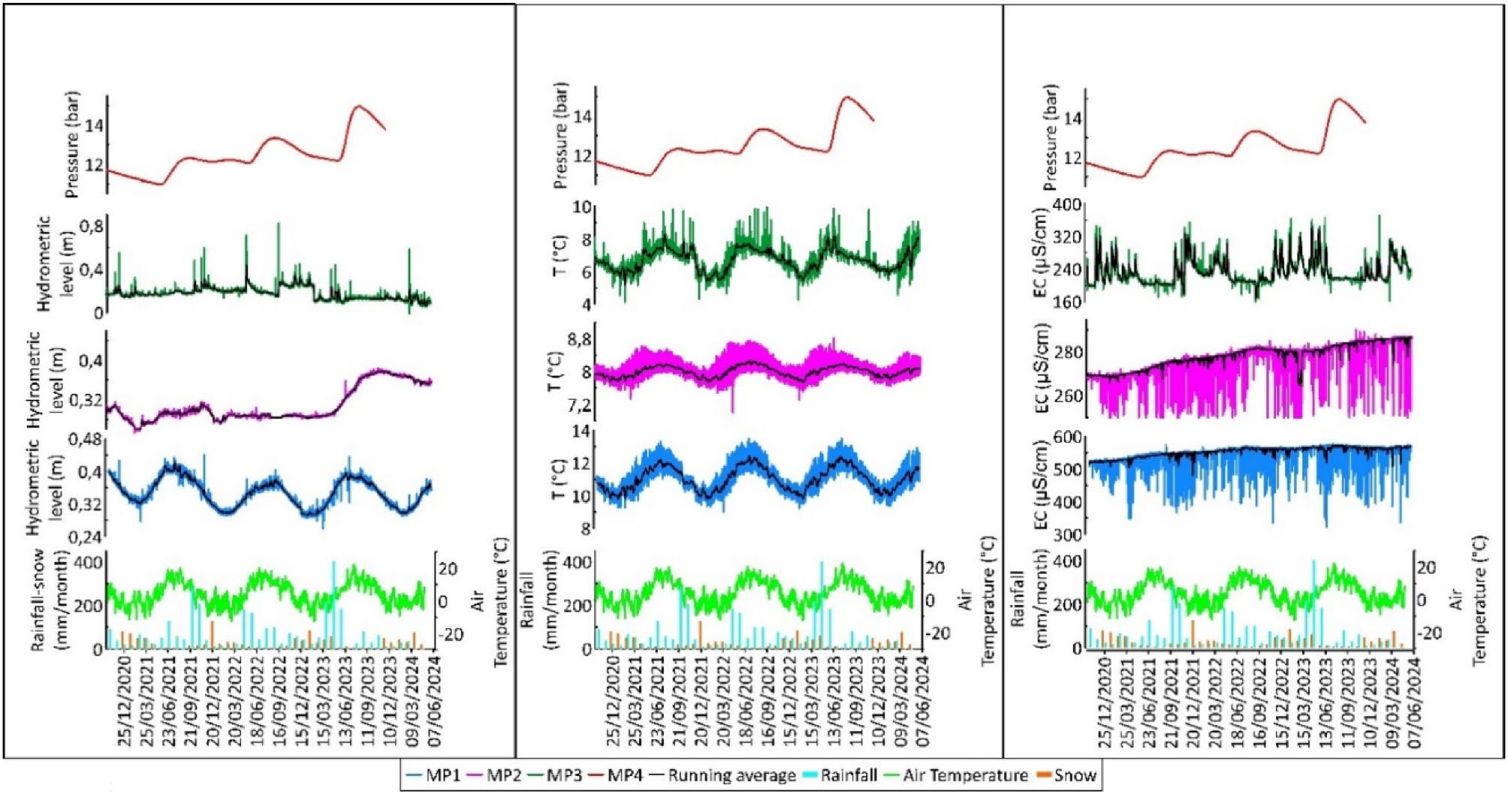
Daily time series of the four stations and the hydrogeological and meteorological data and moving average (thick lines) are also shown; many spikes in Electrical Conductivity are attributed to instrument malfunctioning.

**Fig. 6:**
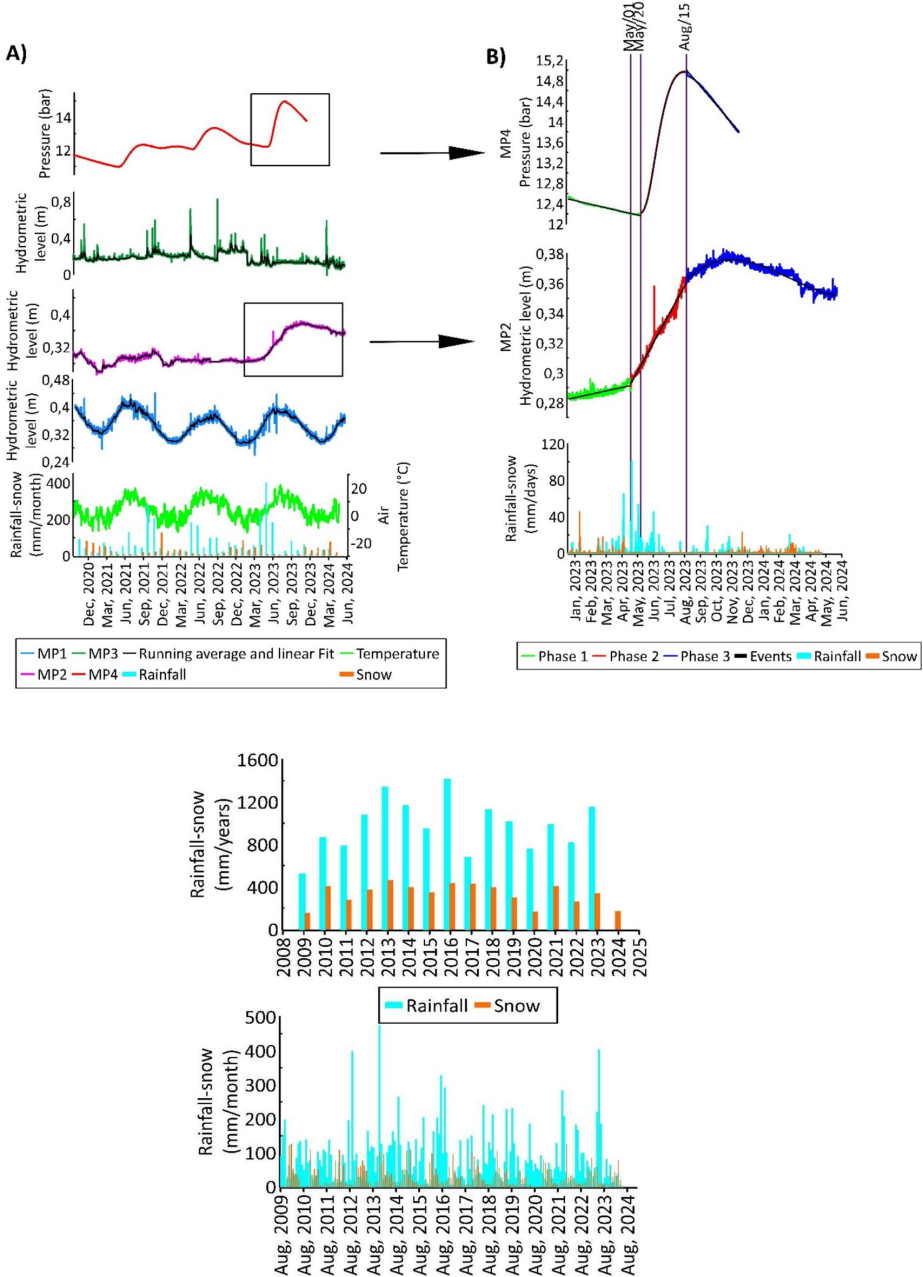
Top: (**A**) Hydrometric level time series and (**B**) time series elaborations. Bottom: Long-term time series of rainfall and snowfall recorded at *Campo Imperatore* gauge station from 2009 to 2024.

The data acquisition is in continuous high-frequency sampling (20 Hz for each channel) of groundwater hydraulic pressure (*Gems sensor*, *3500 series, 0–4 MPa* range), electrical conductivity (www.bc-electronics.it, mod. *Sl-311*) and temperature^33,34^. The meteorological data come from *Servizio Idrografico e Mareografico* of Abruzzi region and they refer to the *Campo Imperatore* gauge (https://www.regione.abruzzo.it/content/idrografico-mareografico). We have collected daily data on rainfall, snowfall (measured as millimeters of water from melted snow), and temperature, spanning the same period as the hydrogeological monitoring (October 2020-June 2024). Figure 5 presents the data time series from the four monitoring stations and the rainfall data from the *Campo Imperatore* gauge. Table 1 provides a statistical summary of hydrogeological data, while Figure 6 shows monthly and annual data for rainfall and snowfall. To filter the instrumental noise, which is particularly evident in the electrical conductivity time series from *MP1* and *MP2*, moving averages are applied to all time series acquired from stations *MP1*, *MP2*, and *MP3*. At *MP1*, minimal and seasonal oscillations in levels and temperatures were detected. Electrical conductivity values remained highly stable, which is supported by the low standard deviation (*STD*) values presented in Table 1. Indeed, *MP1* represents the main discharge area of the Gran Sasso aquifer characterized by long and deep travel within the aquifer, as evidenced by the relatively higher temperature and electrical conductivity values compared to those of the springs fed by the same aquifer (Lorenzi et al.^32^ and reference therein). The springs of the Tirino group (*MP1*) are not influenced by local climatic or anthropogenic factors, showing a limited seasonal oscillation and a decreasing trend in levels over the entire observation period probably due to decreasing in recharge rates (Figure 5^32^). *MP2* (Tempera Spring) exhibits different patterns in seasonal variations compared to the *MP1*. Specifically, while the minimal temperature fluctuations have a seasonal cycle and the electrical conductivity is steady (excluding a slight and negligible increasing trend over the entire period), the variations in water levels are completely different from those recorded at *MP1* (Figure 5). They are characterized by an initial period of invariability, which ends with the onset of a sudden and sustained increase from May 2023 to August 2023. Based on the chemical and isotopic characteristics revealed by previous studies^14,21,22^ the Tempera spring appears to be fed directly from the core of the carbonate massif with rapid transfers likely due to karst circuits that are not entirely known. In accordance with the hydrodynamics characterizing the monitored sites^19^, *MP3*, representing the aquifer core, exhibits a different hydrogeological response compared to *MP1* located at the aquifer SE. In fact, even if the temperature time series for *MP2* and *MP3* show a similar seasonal patterns (see Figure 5), while the time series for water levels of *MP1* with respect to *MP3* show quite different statistical parameters (Table 1)*.* We remark that the electrical conductivity time series of *MP3* is different compared to *MP1* and *MP2*. The monitoring point *MP4* exhibits the most significant variations during the considered period, with an average value of 12.28 bar and a standard deviation (STD) of 0.86 bar (Table 1). This large variability is attributed to the monitoring station’s location at the core of the aquifer, below the recharge area, which allows it to record the greater fluctuations recorded by the saturated part of the aquifer. It is also possible to observe seasonal oscillations in this case, although they are not as well modulated as those observed at *MP1*. Over the monitoring years, the pressure has shown an increasing trend, with the highest value of around 15 bar recorded in August 2023.

**Table 1:**
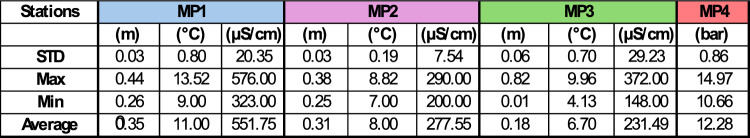
Statistical synthesis of hydrogeological results.

Table 2 and Figure 6 show the monthly and annual data on rainfall and snowfall recorded at the *Campo Imperatore* station during the monitoring period. The rainiest month was May 2023, with 402.6 mm of cumulative rainfall, while the snowiest month was December 2021, with 126 mm of snow. 2023 was the year of highest rainfall during the considered period (October 2020-April 2024).

**Table 2:**
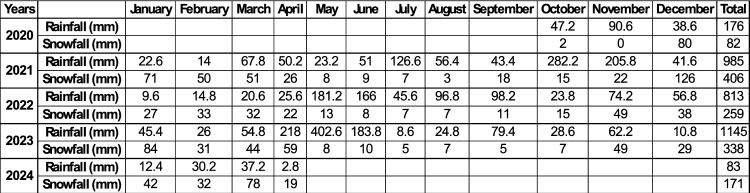
Monthly cumulative values of rainfall and snowfall recorded at *Campo Imperatore* gauge station from October 2020 to April 2024.

With the aim of verifying what occurred during the night between August 14 and 15, 2023, the hydrogeological data recorded before, during, and after the acoustic *bang* event will be analyzed below. The focus is primarily on the time series of hydrometric levels recorded at the four stations (Figure 6A). The time interval considered is a zoom of the longest period considered before, spanning from January 1, 2023, to May 29, 2024. The analysis focused on stations MP2 and MP4 because their data showed a strong correlation with the aquifer’s core zone, a finding consistent with previous research. In fact, the time series of *MP1* and *MP3* showed no significant variations from the August 14, 2023, event, suggesting they are more affected by the aquifer’s regional cycles. Conversely, at *MP2*, a rapid increase in hydrometric levels has been observed since May 1, 2023 (*MP2*, Phase 2, in Figure 6B).

The slope of the incremental segment varies and decreases sharply on August 15, 2023 (*MP2*, Phase 3, in Figure 6B). Similarly, the pressures recorded at *MP4* are characterized by a rapid increase from May 20 to August 15, 2023 (*MP4*, Phase 2, in Figure 6B), followed by an equally sudden decrease (*MP4*, Phase 3, in Figure 6B). The increases in levels and pressures at stations *MP2* and *MP4*, which began in May 2023, follow intense rainfall events, as recorded by the *Campo Imperatore* station. It should also be noted that May 2023 was the rainiest month of the entire period considered (Table 2). Additionally, with 402.6 mm of rainfall, May 2023 ranks as the second wettest month since August 2009, following the 473.4 mm recorded in November 2013 (Figure 6, *bottom*).

The recharge of the Gran Sasso aquifer, as known from the literature^21^^,^^32^, is strongly linked to the snow recorded in the endorheic basin of *Campo Imperatore*, which, when melting, feeds the basal aquifer. However, it is recognized that the quantity of snowfall of the Gran Sasso, among other areas (Lorenzi et al.^32^ and reference therein) has decreased in recent years^32^. On the other hand, rainfall does not show the same trend,instead, there is an increase in the intensity of spring precipitation, as observed in spring 2023. This can potentially affect the recharge mechanisms of fractured carbonate aquifers, which are also subject to intense karstification processes, such as the Gran Sasso aquifer. The sharp hydrometric levels increase observed at *MP2* and *MP4* since May 2023 could therefore be the result of the activation of the karst circuits of the massif following exceptional rainfall. The absence of hydrometric level variations at station *MP1* is also consistent with the observation that this station is fed by a longer, slower, and deeper basal circulation. This occurs primarily within the fractured medium of the carbonate aquifer rather than through the rapid flow associated with the karst conduits that affect stations *MP2* and *MP4*.

### Angular rotation recorded by the gingerino ring laser gyroscope

In correspondence of the strong *bang* happened on August 14, 2023 (22:00 UTC), *GINGERINO*’s data shows a very large signal, as the other multi-parametric environmental monitors. Due to the exceptional nature of this event, we have investigated the general functioning of the apparatus in an extended period, finding that, since approximately May 10, 2023, signal amplitudes were anomalous, and that the whole apparatus was not operating in optimal condition. We verified the correct performance of the *Ring Laser Gyroscope GINGERINO* (*RLG*) by checking that events recorded by *GIGS* were correctly detected.

In the following, the *RLG* will be briefly described, and its data analysis will be reported, divided into two main time spans:

(i). The first part is dedicated to the period of August 11–15, 2023, with the goal of also showing the high-frequency components of the event;
(ii). The second part reports five months of data from May 1 to September 22, 2023, with the aim of analyzing the instrument’s behavior over a long period that includes the large *bang* event.

#### GINGERINO: Apparatus and angular rotation reconstruction

Large area *RLG* have the objective of measuring the Earth angular velocity, with unprecedented sensitivity. They provide exceptional insights for geophysics in general because they enable the local measurement of the angular rotation rate on the Earth’s crust, with a bandwidth up to the kHz range, and can resolve daily and sub-daily variations^35^–^44^. The same device is able to measure large and very tiny signals, i.e. data valuable for rotational seismology, geodesy, and fundamental physics tests^45^^–51^. For further details on rotational seismology, see: https://www.rotational-seismology.org/.

**Fig. 7:**
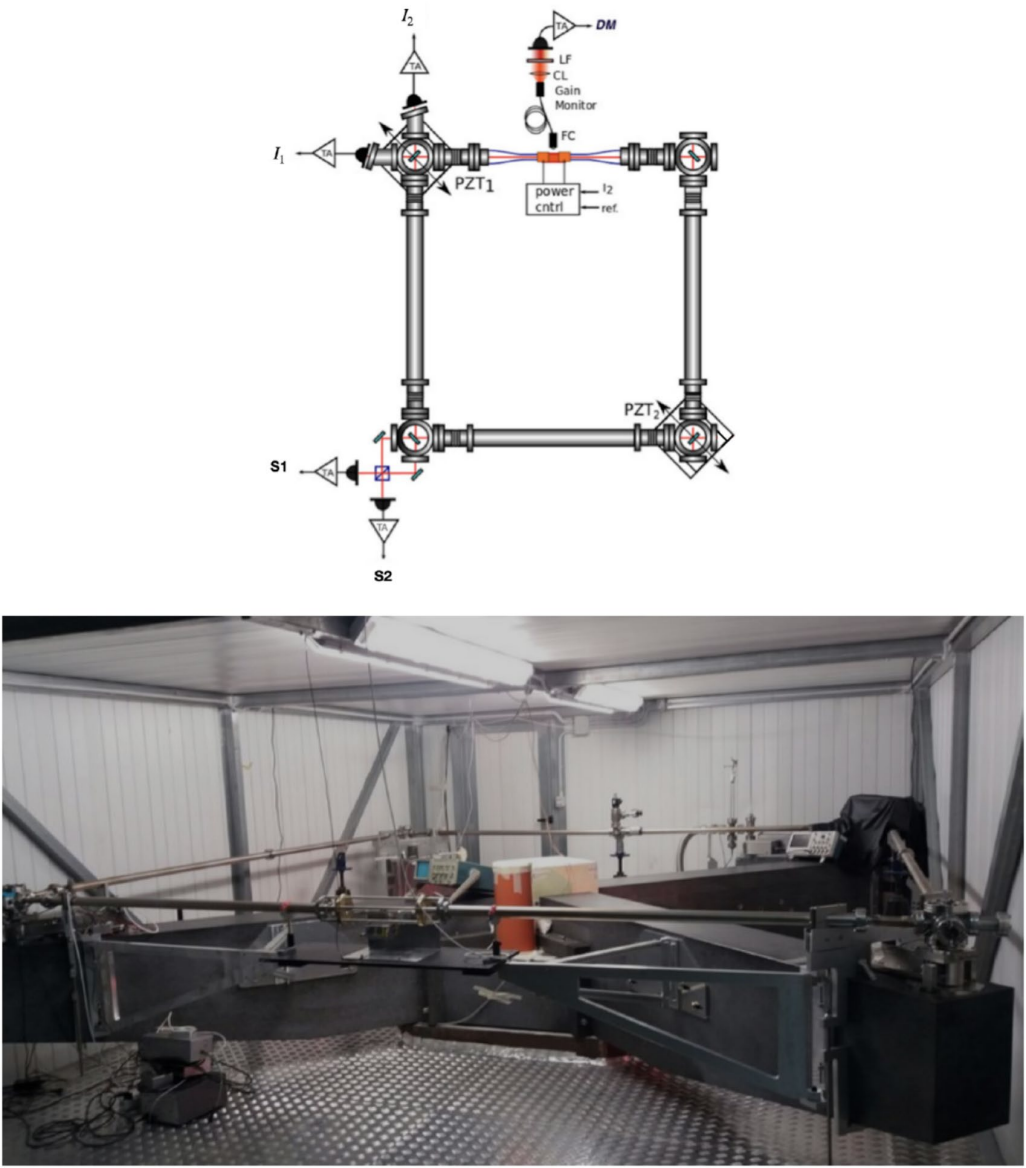
Top: Schematic view of a *Ring Laser Gyroscope* (*RLG*); in particular, 2 beat note signals (S1 and S2), and the intensities of the single laser beams (I1 and I2) are acquired. Bottom: The *GINGERINO RLG* at *LNGS-INFN*; the *GIGS* broadband seismometer is co-located in a yellow box at the center of the *RLG* diagonals.

The working principle is the Sagnac effect: Two counter-propagating light beams traveling in a closed ring path complete the round trip with a difference in time proportional to the absolute (or inertial) angular rotation rate of the path. Several devices have been developed based on the Sagnac effect; so far, the most sensitive one is the *RLG*, based on an optical cavity in which an active medium is inserted, in order to generate the two counter propagating light beams inside the closed path. In this case, the frequency of the two counter-propagating beams are slightly different because the Sagnac effect and the interference of the two beams transmitted by the cavity results in a beat note signal, in the ideal case is the Sagnac frequency ${f}_{s}$:

1 $${f}_{s}= 4 \frac{A}{P\lambda } \bullet \Omega \mathrm{cos}\theta$$

where *A* is the area delimited by the light path, *P* the perimeter, *Ω* the absolute value of the angular velocity of the optical cavity, *λ* the laser wavelength and *θ* is the relative angle between the direction (or rotation axis) of Ω and the area vector **A** of the closed path; ${f}_{s}$ is expressed in Hz, in the following ω_*s*_ in cycle per second will be used, keeping in mind that ω_*s*_ = 2 π ${f}_{s}$. For *RLG* attached to the Earth crust, as *GINGERINO*, Ω is the Earth rotation rate plus all local rotations. According to Eq. 1, a change in $\theta$ would lead to modify ω_*s*_. In general, with a single *RLG* it is impossible to distinguish between changes in cavity rotation rates or inclination. Data from a co-located tiltmeter, mounted on top of the *RLG* monument, is used to determine the inclination. *GINGERINO* is a *RLG* prototype operative inside *LNGS-INFN*, see Figure 4; it is constituted by a He-Ne Ring Laser (λ ∼ 633 nm) based on a square optical cavity (3.6 m in side), containing as active medium a He-Ne gas mixture. The optical cavity of *GINGERINO* is horizontally oriented (i.e. vertical area vector) with beat-note mean value around 280 Hz. A *RLG* schematic view and a picture of *GINGERINO* are shown in Figure 7. A full description of the *GINGERINO* apparatus can be found in Di Virgilio et al.^52^ and^53^.

*GINGERINO* runs unattended for months, and routinely every 3–4 months a team of two people accesses the area and operates to restore alignments of the cavity and to upgrade electronics or the optical set-up. In 2023, the first intervention was in February; in May, a new operation has been done to upgrade the discharge electronics and to restore the optimal alignment. The gyroscope operates unattended, recording data that is then analyzed if a significant earthquake occurs. The signal is coming from the interference of the two counter-propagating beams transmitted by one of the cavity mirrors. The interference signal contains small perturbations induced by mirror imperfections and by the non-linear dynamics of the laser. To reconstruct ω_*s*_^54^^,^^52^, the two output beams interference and the diagnostics of the two counter-propagating light beams (mono beams) intensity (Figure 7, *top*) are used. The analysis procedure removes instrumental disturbances induced by the laser itself, due to the backscatter on the mirrors and to the non-reciprocity of the radiation paths in the optical cavity (null-shift systematics). We indicate with ω_*m*_ the frequency measured from the raw signal, ω_*s0*_ the processed signal where the backscatter noise has been eliminated, and ω_*s*_ the signal free from backscatter and null shift systematics. In the correction procedure, the polar motion, estimated at the *GINGERINO* latitude and longitude by the International Earth Rotation and Reference Systems Service (*IERS*), and the tides, using the list elaborated with the program *GOTIC2_mod*, are taken into account. The interested reader can find more details about the analysis procedure in previous papers^52,53,55^.*GINGERINO*^40,56,57^ is free running and the laser modes change, causing mode jumps, and split mode operations. The first case appears as a fast transient of fraction of second duration. In the second case, the Sagnac signal has a frequency in the MHz range, and our data acquisition system cannot acquire it. Split mode operation can last for several hours and corresponding data are lost. To select the relevant data for the analysis, portions with split mode operation or those affected by mode jumps are eliminated,in normal typical conditions, more than 90% of the data are kept.

#### Analysis of *GINGERINO* data at high frequency bandwidth and comparison with co-located tiltmeter and seismometer

**Fig. 8:**
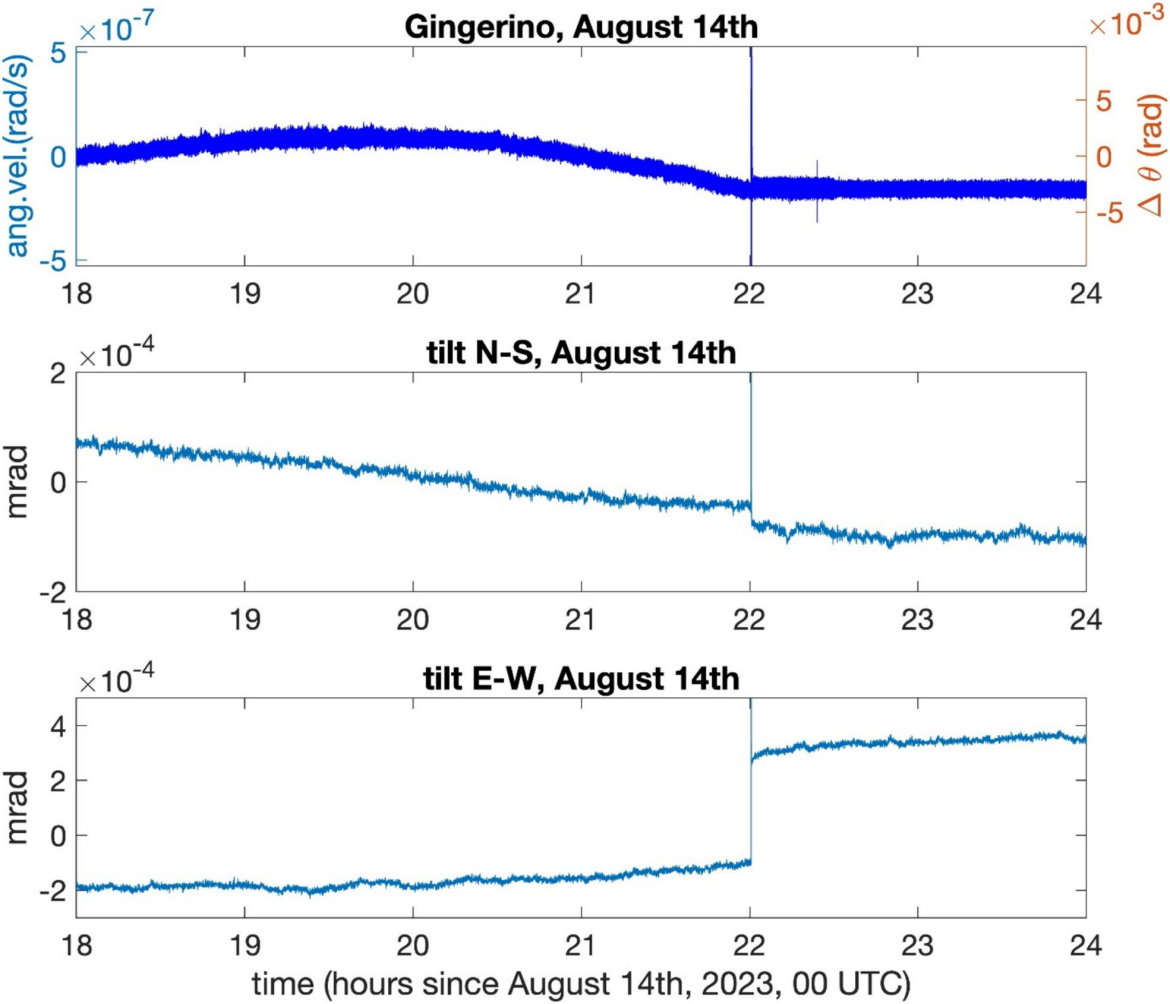
*GINGERINO* and tiltmeter signals recorded on August 14, 2023. The occurrence of the *bang* event around 22:00 UTC is clear in all shown data. Assuming the *RLG* signal affected solely by a change Δ $\theta$ of the instrument inclination, the right axis in the top panel demonstrates that such a change is not compatible with tiltmeter data, since it would correspond to a change more than one order of magnitude larger than the one recorded by the tiltmeter in the N-S direction. Because its area vector is vertical, *GINGERINO* is less affected by changes in its E-W inclination.

**Fig. 9:**
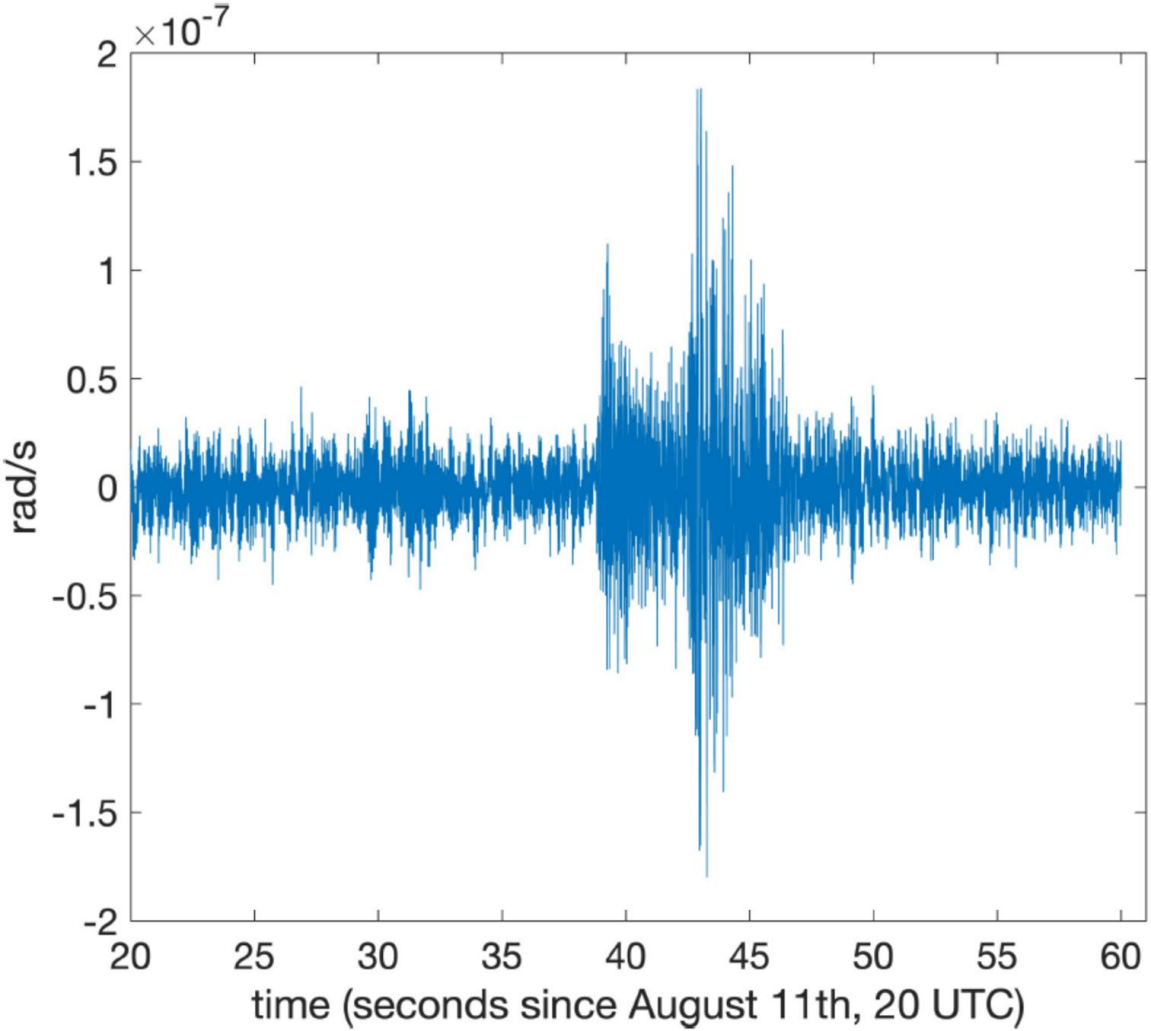
The small seismic event registered by *GINGERINO* at the end of August 11, 2023; *P-* and *S-* wave arrival times show a difference of about 3.5 s. This earthquake was localized by the National Seismic Network of *INGV* about 24 km away from *LNGS-INFN* at a depth of 11 km, with magnitude *M*_*l*_ 2.1, in a WNW direction, compatible with the *S-P* arrival time difference recorded by *GINGERINO* (https://terremoti.ingv.it/event/35800831?timezone=UTC).

The analysis presented in the following, carried out with high frequency bandwidth, considers the interval 11–14 August 2023, and uses the *ω*_*s*_ or *ω*_*s0*_ indifferently, since they are equal at a level better than 1%. Figure 8 shows plots of the *GINGERINO* and tiltmeter signals on August 14, 2023 (DOY: 226). In correspondence to the *bang*, the tiltmeter shows a sharp 0.4 μrad inclination change in the East-West direction, providing a clear fracture evidence. Comparison with tiltmeter data suggests that *GINGERINO* signals were mostly affected by variations in the angular rotation rate since, otherwise, they would correspond to changes in inclination more than one order of magnitude larger than those observed by the tiltmeter. Assessment of the *RLG* operation is found by analyzing its ability to record a small local seismic event occurred on August 11, three days before the *bang* event. Figure 9 shows detail of the corresponding *GINGERINO*, observed by the co-located tiltmeter and broadband seismometers (*GIGS*).

**Fig. 10:**
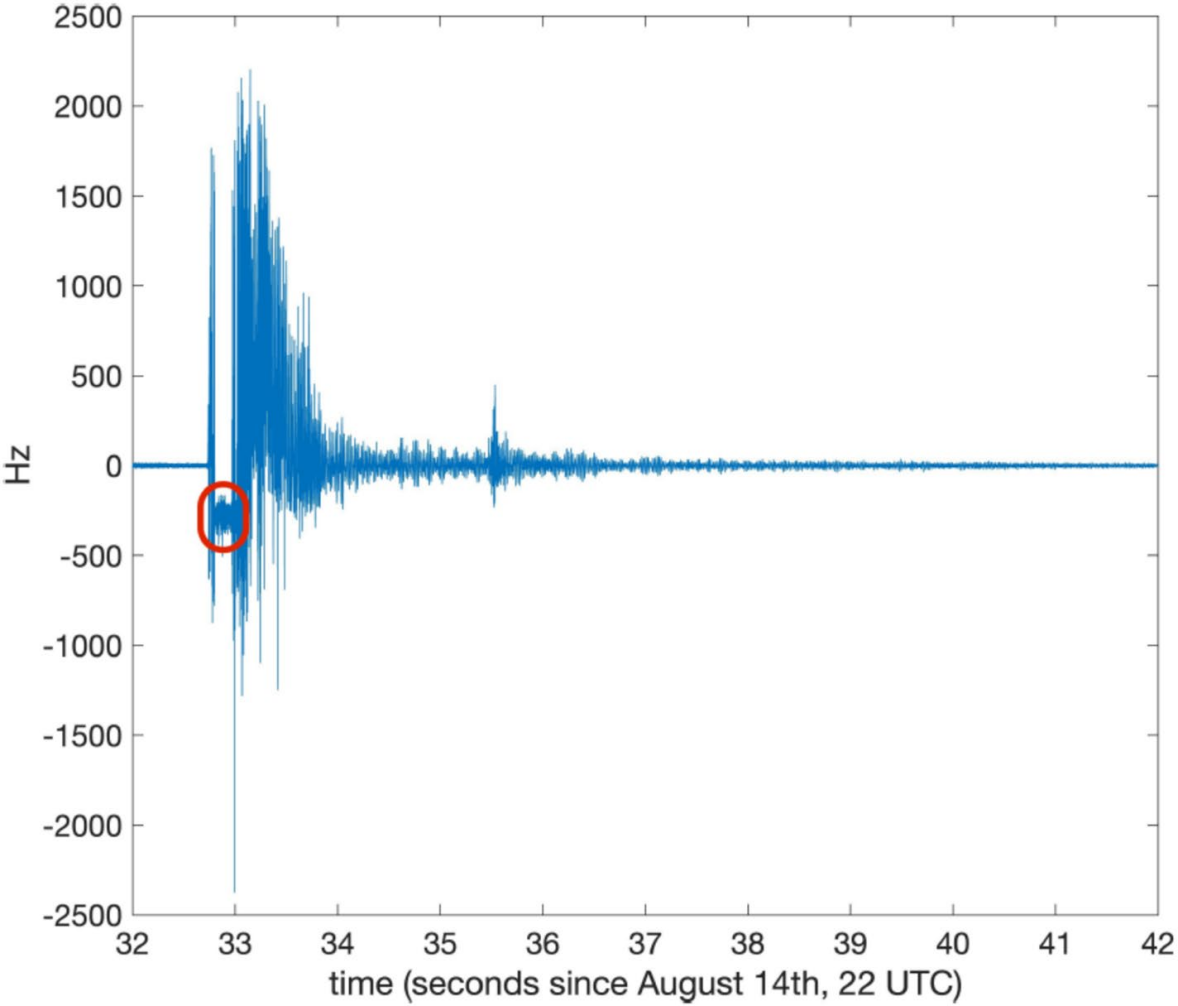
*GINGERINO* signal corresponding to the mountain *bang* event on the night of August 14. The data between seconds 32 and 33, circled in red, has no physical meaning, since at that time the laser was switched off by the violent *bang* event.

Figure 10 shows the signal for the *bang* event. One hour of data collected at a 5 kHz bandwidth, without filter or decimation, were considered. Frequency components above 1 kHz appear in the spectrum. Due to the strong signal from the bang event, the laser was shut off, causing a gap between seconds 32 and 33. The resulting portion of the measured frequency, encircled in red in Figure 10, has no physical meaning.

**Fig. 11:**
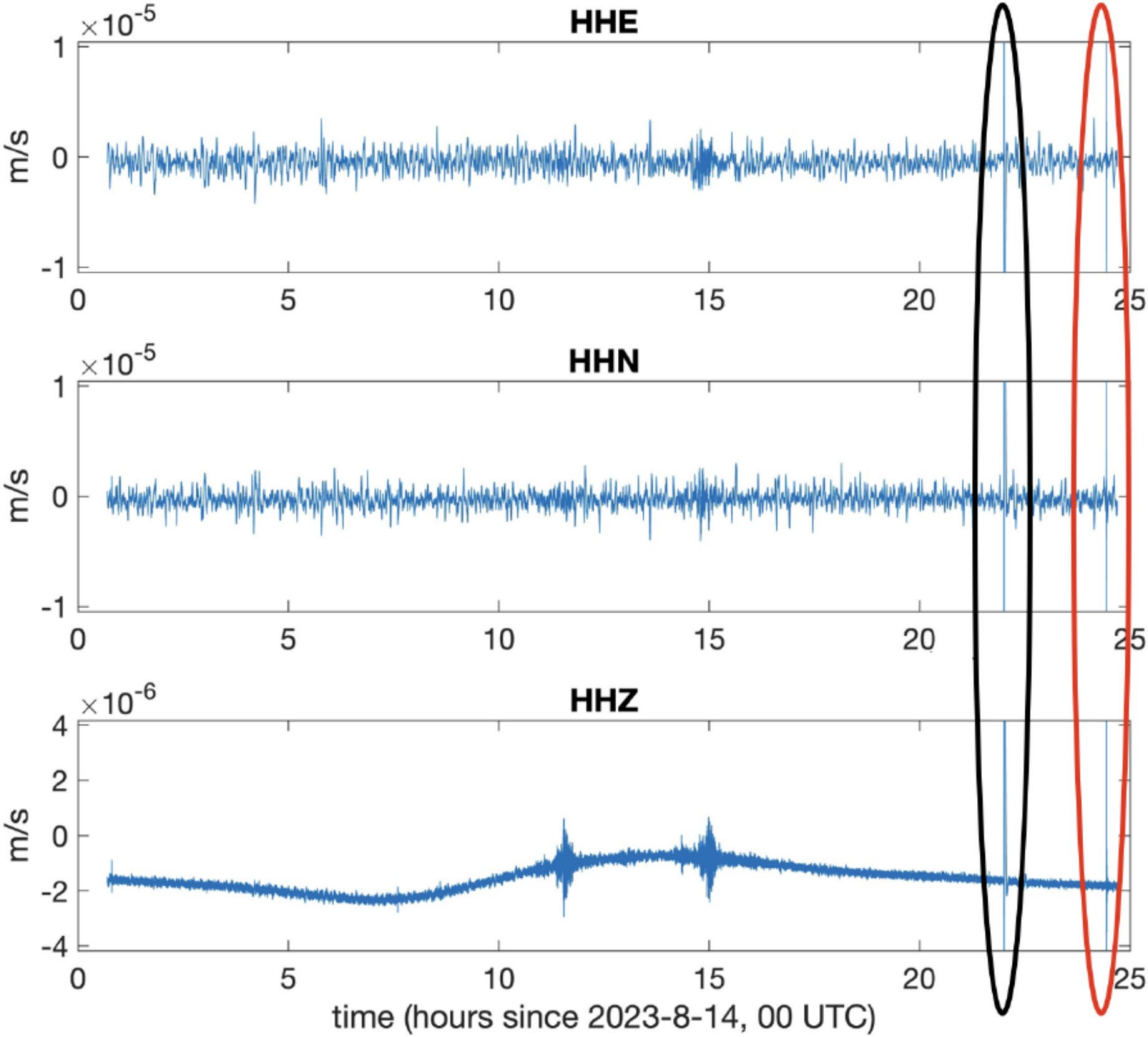
*GIGS* broadband waveforms for the day of August 14, 2023 (*HHZ, HHN* and *HHE* are the vertical, North-South and East-West components respectively); the black area highlights the occurrence of the *bang* event while the red area shows a micro-earthquake which was not localized by seismic monitoring network (see Figure 12 for detail); this small event (*M*_*l*_ ~ 1) presents the same time arrival difference of the *P-* and *S-* waves (about 3.5 s) as the August 11 event (see Figure 9).

**Fig. 12:**
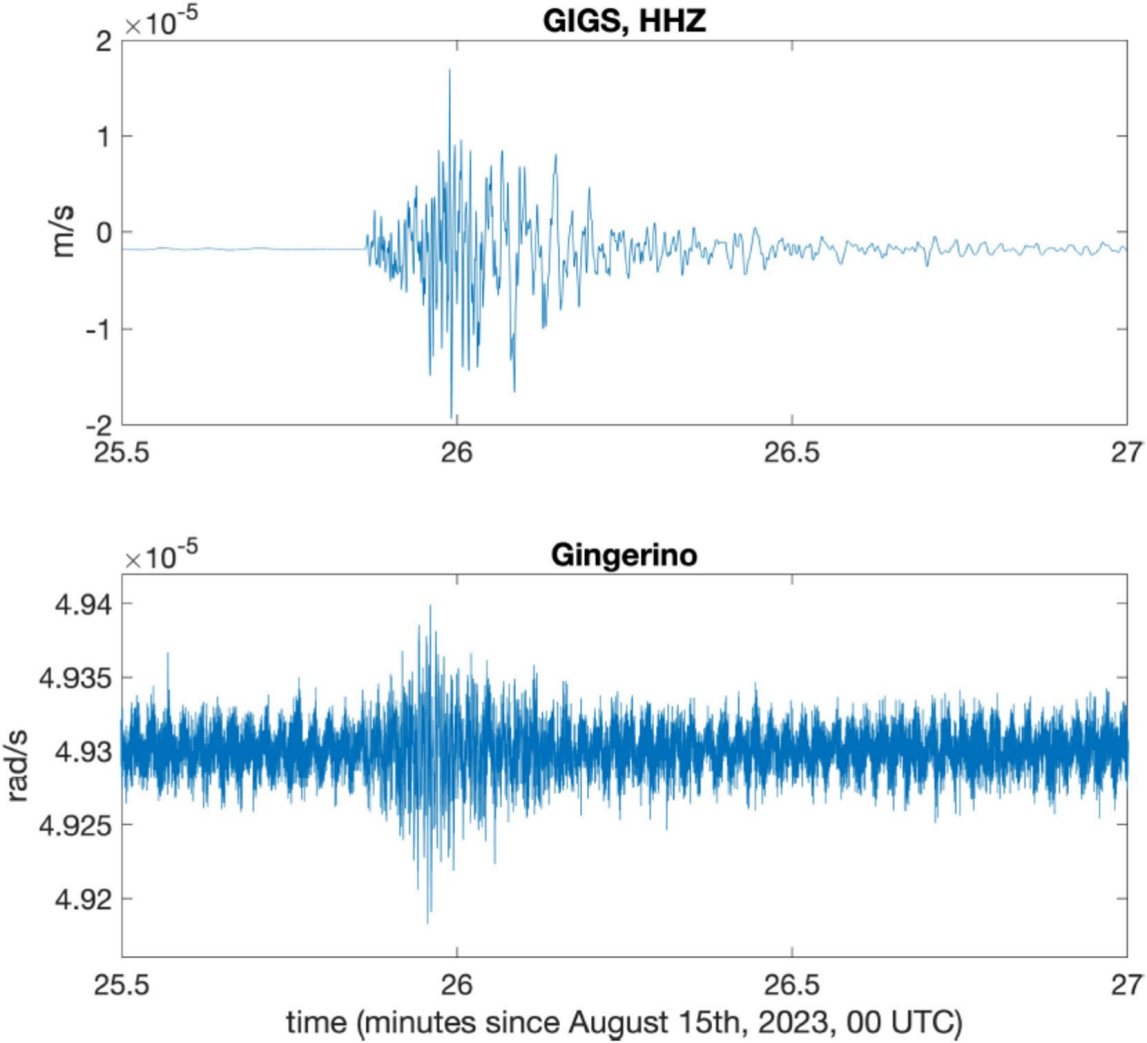
Detail of the local micro-seismic event of August 15, 2023, 00:26 UTC as recorded by *GIGS* (*HHZ*: vertical component) and *GINGERINO*. the duration magnitude (*M*_*d*_) can be calculated using the relationships in Castello et al.^58^, and Casale and Mazza^59^, obtaining a value of *M*_*d*_ = 1.0.

Figure 11 shows the *GIGS* 3-components signals in a period starting from August 14, 2023, 00:00 UTC, up to the first hour of August 15, 2023. The large signals circled in black represent a signature of the *bang* event, the one circled in red is a small seismic event; this micro-earthquake was not localized by seismic monitoring (*M*_*l*_ ~ 1) and presents the same arrival time difference between P- and S- waves (about 3.5 s) as the August 11 event shown in Figure 9. Therefore, it likely to be an aftershot. The duration magnitude (*M*_*d*_) can be calculated using the relationships in Castello et al.^58^ and Casale and Mazza^59^, obtaining a value of *M*_*d*_ = 1.0. In Figure 12, this small seismic event is shown, as recorded by *GINGERINO* and HHZ *GIGS* (vertical component). The well known typical relaxing time, called *whale*^60^^,^^61^ has been observed in the *GIGS* sensor (∼ 240 s that is the natural period of sensor) after the violent hit,this is useful to define the duration of the signal which can be evaluated under 10 s, corresponding to a duration magnitude *M*_*d*_ close to 0.6.

The *coherence* function, a spectral analysis tool used to characterize the frequency content of a signal, was computed using *MATLAB*’s *mscohere* function. Such analysis, based on the *magnitude squared coherence* is a measurement of the correspondence in normalized magnitude of two physical quantities, at various frequencies. Coherence analysis was performed using *GIGS* and *GINGERINO* to detect correlations between corresponding signals. Coherence was investigated before, during, and after the *bang* event. Before the event, the coherence is negligible. Remarkably, the mountain *bang* signals registered by the broadband *GIGS* sensor have several frequencies in common with the rotational *GINGERINO* signal, in particular the channel *HHN*, North-South vibrations, where coherence shows a magnitude around 0.4 in the frequency band 10–35 Hz. A magnitude below 0.1 was found for all other data.

**Fig. 13:**
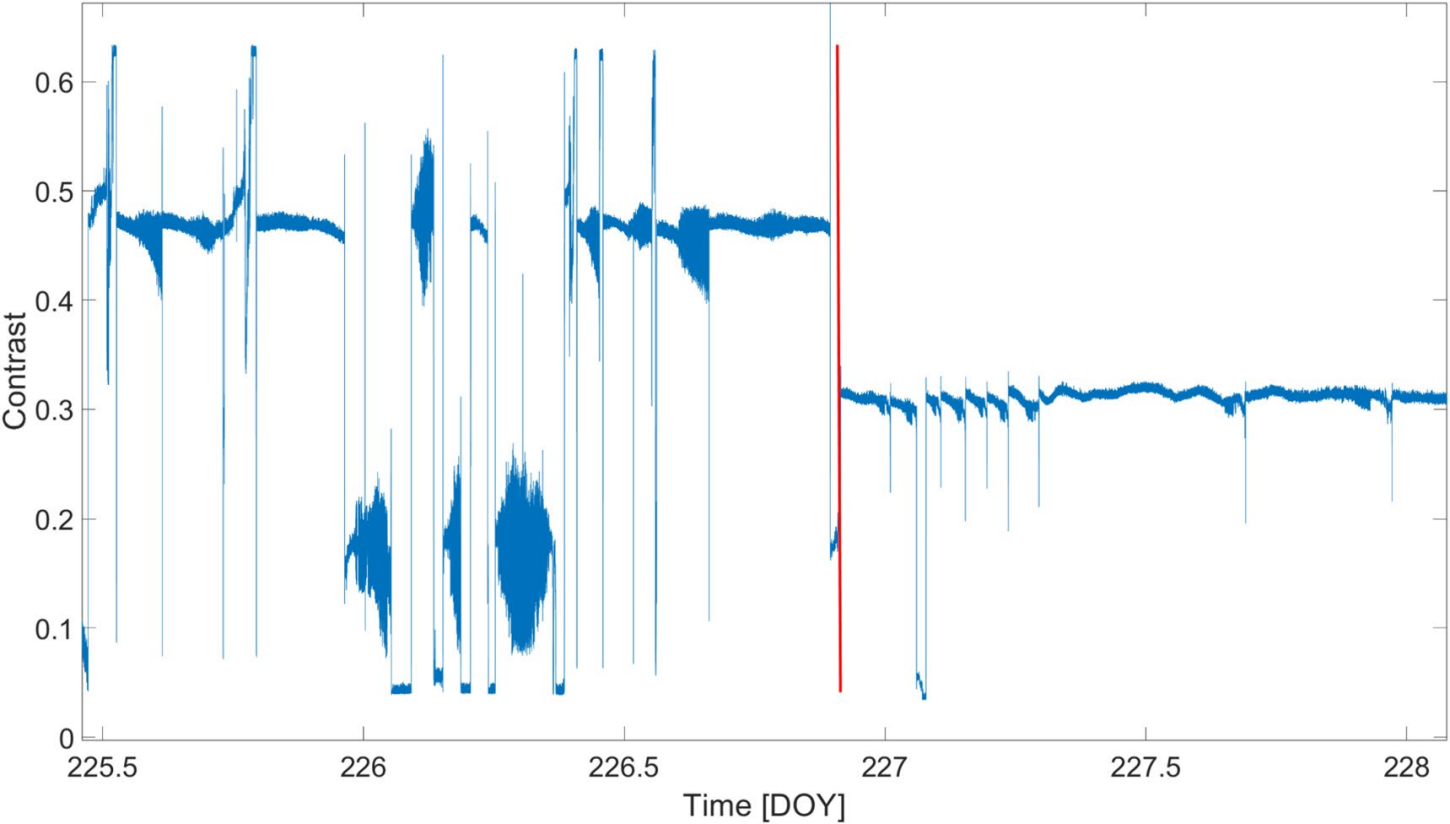
Fringe contrast *C* of the *GINGERINO* beat note during August 13–15, 2023 (corresponding to DOY 225–227); the X-axis represents the day of the year (DOY); the red vertical line indicates the occurrence of the *bang* at 22:00 UTC on August 14 (DOY: 226). After the *bang* the contrast *C* drops below 0.3, the beat note signal is in any case present, and signals can be well reconstructed, see Figures 9 and 12, but with larger measurement noise. In the analysis the portions with Contrast sharply decreases are removed, since the beat note is not present or the laser is not correctly operating.

An additional figure of merit to describe the operation of the *GINGERINO* is the contrast (*C*), usually called fringe contrast or visibility, used in interferometry to quantify the quality of the interference, the maximum contrast being 1. The *RLG* signal is a beat note, i.e. a sinusoidal signal, whose amplitude is in between two boundaries: the maximum (*MAX*) and minimum (*MIN*). Accordingly, the contrast is defined as *C* = (*MAX*+*MIN*)/(*MAX*-*MIN*)^62,63^. Since contrast *C* depends on the relative positions of the mirrors defining the interferometer cavity, its investigation can reveal the occurrence of large external disturbances leading to mechanical misalignments of the cavity. The behavior is shown in Figure 13: After the *bang* event, *C* tends to lower values, which, however, remain almost constant on time. In other words, the *bang* event produced an abrupt variation of *C*, followed by a recovery to almost stable values. In ordinary operating conditions, *GINGERINO* shows a typical contrast above 0.8, larger than the values obtained after the *bang* event. We remark that, despite the loss in contrast, the ability to record seismic data remained unaltered, as demonstrated by the already mentioned seismic event in Figures 9 and 12.

**Fig. 14:**
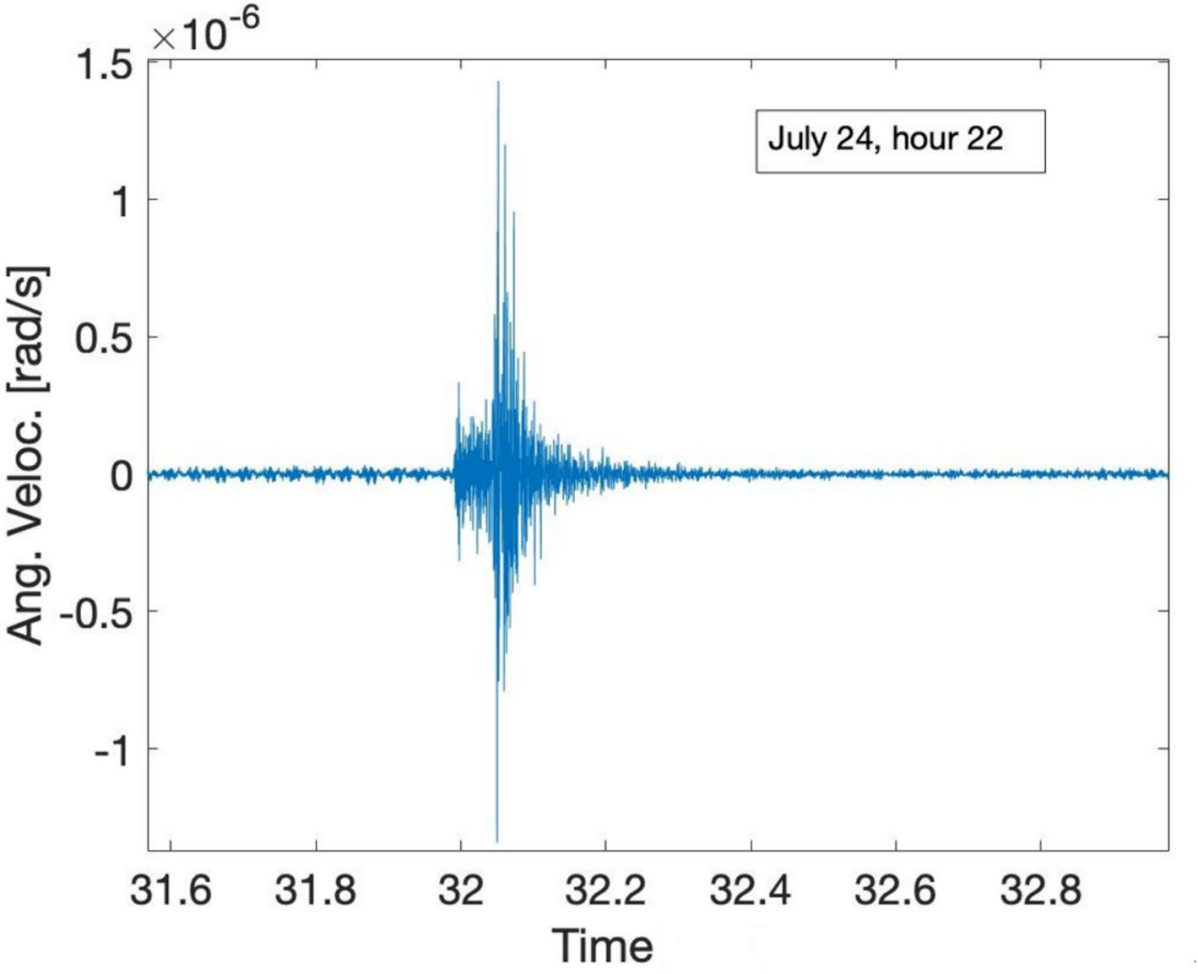
Local earthquake of July 24, 2023 seen by *GINGERINO* with magnitude *M*_*l*_ 3.0 which occurred at 22:31:55 UTC at a distance of approximately 20 km in a Westerly direction. The axis time is in minute; the waveform is very clear for more than 30 s and the arrivals of the *P-* and *S-* waves are clearly visible.

To further test the validity of *GINGERINO* data, we have looked at local earthquakes. Figure 14 shows the one occurred on July 24 at 22:31:55 UTC, with magnitude *M*_*l*_ 3.0 (https://terremoti.ingv.it/en/event/35640851?timezone=UTC) at a distance of approximately 20 km in a Westerly direction; the arrivals of the *P-* and *S-* waves are clearly visible.

### Analysis of GINGERINO data from May 1, 2023

Analysis of the high frequency bandwidth signals reported in the previous section suggests that *GINGERINO* clearly showed evidence of the *bang* event, in particular in the strong enhancement of the recorded signal that also displayed coherence with the *GIGS* broadband seismometer components. However, the nature of the local ground motion leading to the observed signal features cannot be inferred from *GINGERINO* data. As a matter of fact, rotation of the *RLG* cavity induced by the *bang* event was accompanied by some mechanical misalignment, whose effects, according to the contrast *C* analysis remained also after the event. It is certainly possible to say that the external perturbation due to the *bang* event was much higher that typical situation.

**Fig. 15:**
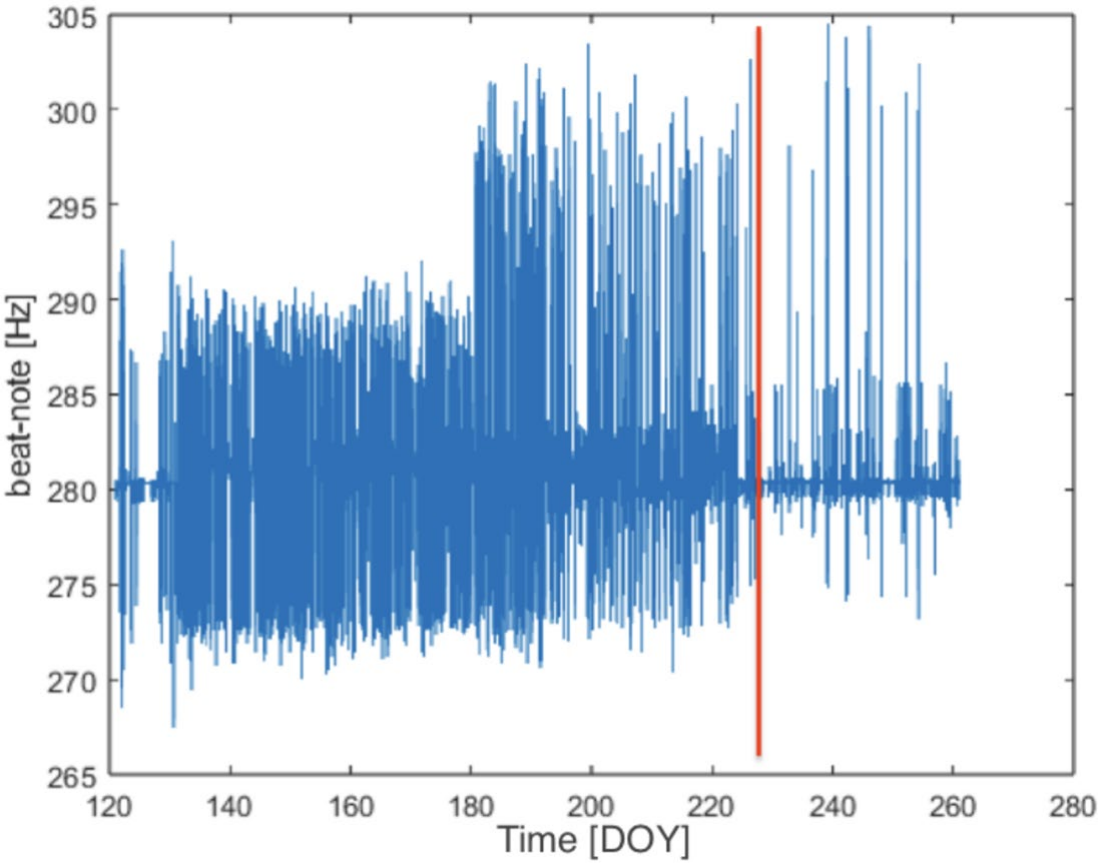
The recorded beat-note *ω*_*m*_, plotted at 5 Hz sampling rate without any selection; the X-axis represents the day of the year (DOY); the red vertical line indicates the occurrence of the *bang* at 22:00 UTC on August 14 (DOY: 226).

Now we focus on the analysis of *GINGERINO* data from May 1 (DOY: 121) to September 20 (DOY: 263) of 2023. The signal is reconstructed here using the standard procedure for the evaluation of the three levels (*ω*_*m*_, *ω*_*s0*_ and finally *ω*_*s*_). The final one, *ω*_*s*_ is obtained by subtracting the null shift disturbances related to the non-linear dynamics of the laser. A similar procedure was adopted for a comparison between *GINGERINO* and *GNSS* (Global Navigation Satellite System) data^50,56^. In general, a linear regression procedure was applied on data corresponding to three consecutive days, keeping the central day, while in the present analysis the linear regression was done every 18 hours, keeping only the central 6 hours. For reference, Figure 15 shows the measured beat note *ω*_*m*_,despite data shown here are raw and no selection is applied,it is clear that between day 130 (May 10) and 226 (August 14) of year 2023 the response is particularly noisy.

**Fig. 16:**
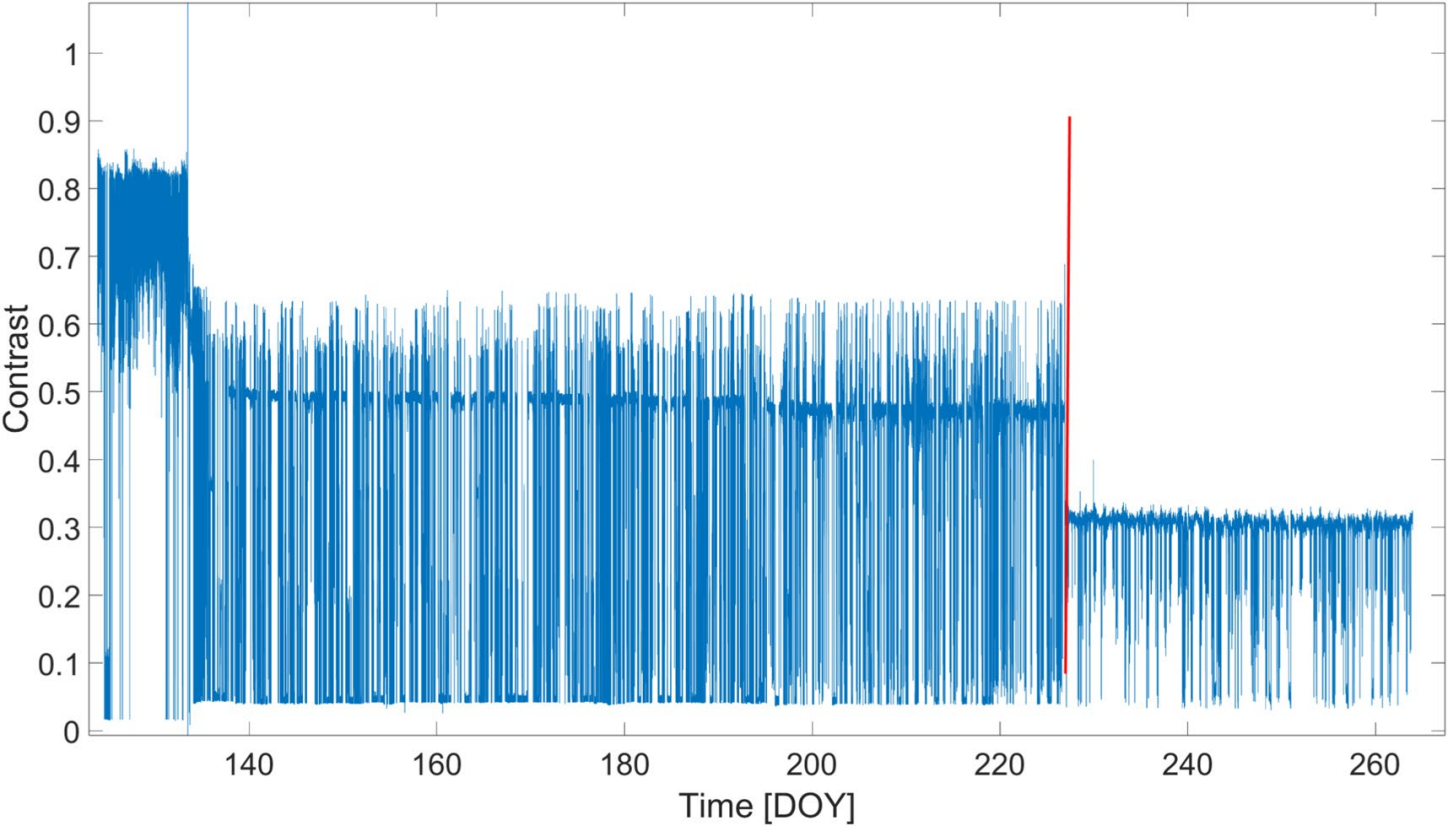
Fringe contrast (*C*) of the recorded beat note, from May to September 2023; the X-axis represents the day of the year (DOY); the red vertical line indicates the occurrence of the *bang* at 22:00 UTC on August 14 (DOY: 226). Several effects must be distinguished in this figure: Very often, *C* approaches zero for a short period, mainly due to laser problems (split-mode operation and mode jumps), and the corresponding data are removed from the analysis; *C* slowly degrades, starting from the maximum when the apparatus is realigned, and these two effects are always present when several months are combined. However, the figure shows that around May 11 (DOY: 131) and the time of the *bang*, the contrast drops sharply, effects caused by external perturbations strong enough to misalign the cavity and the readout optics, degrading the quality of the interference of the two beams. With a low *C*, the measurement noise increases and the measurement is more influenced by variations in the light entering the detector and not due to the interference.

A data selection procedure is typically needed in *RLG* to remove data affected by mode jumps and the laser’s split mode operation. For this purpose, the fringe contrast *C* was continuously evaluated; Figure 16 shows the *C* values for the entire period considered. Data corresponding to very low *C* values are generally neglected in the analysis, although additional diagnostic signals, including the mono-beam intensities, are also taken into account.

We underline again, how, owing to the optical interference nature of the *RLG* signals, a strong dependence on the optical component alignment is found. Since, as already mentioned, operation of the *RLG* was not in optimal conditions in the considered period and, moreover, the perturbation following the *bang* event affected the alignment, we introduced additional selection criteria. They were based on the difference (*RES*) of the frequencies reconstructed from the two beat note signals available in our setup (*S*_*1*_ and *S*_*2*_ in Figure 7, *top*), that allowed to account for external noise injected into the cavity. Once selection has been carried out, selected data are averaged and resampled down by a factor 300, in order to have approximately one point per minute.

**Fig. 17:**
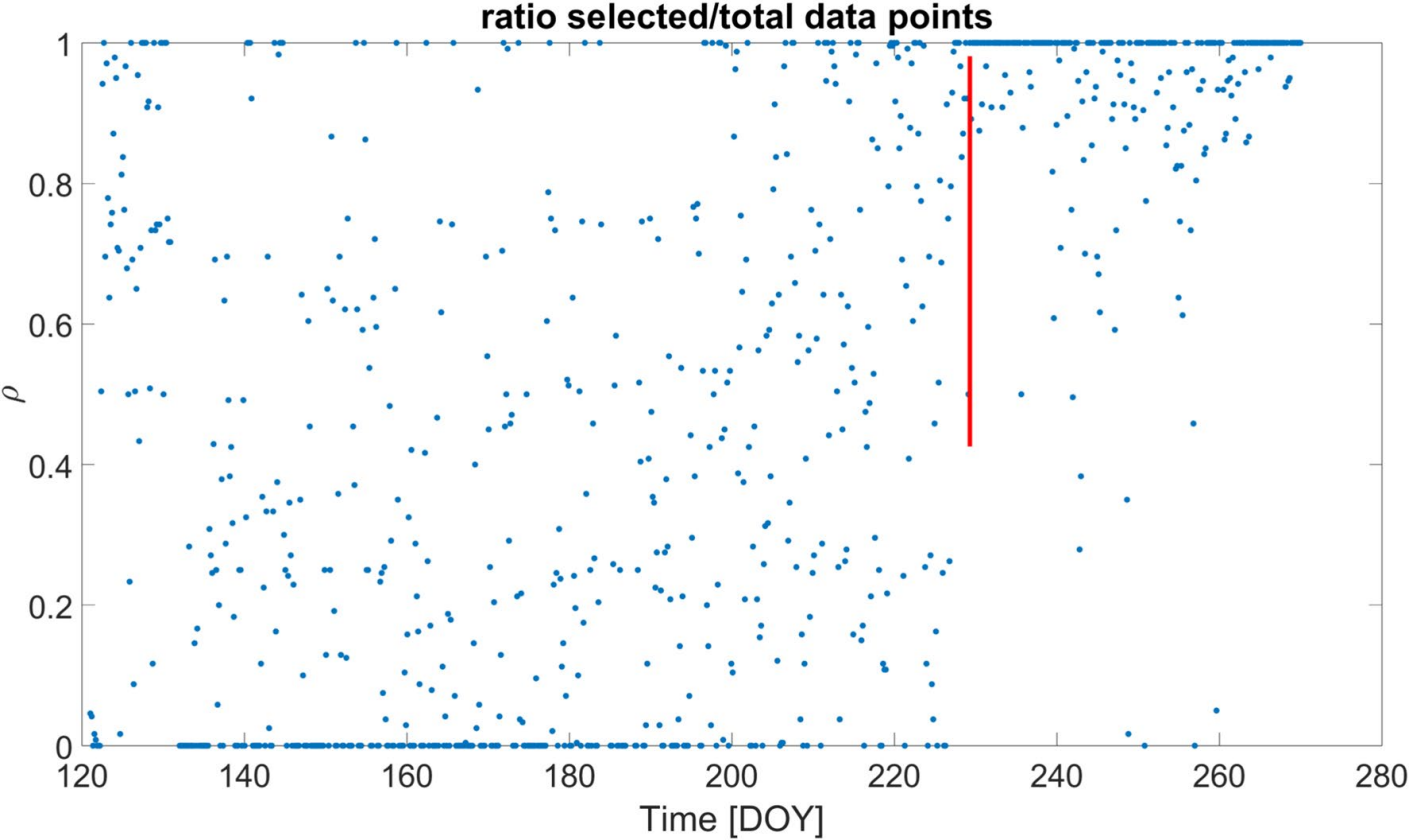
Ratio (*ρ*) of the selected over total data points considered in the analysis in the period ranging from May to September 2023; 65% of data are selected over this period; the X-axis represents the day of the year (DOY); the red vertical line indicates the occurrence of the *bang* at 22:00 UTC on August 14 (DOY: 226).

**Fig. 18:**
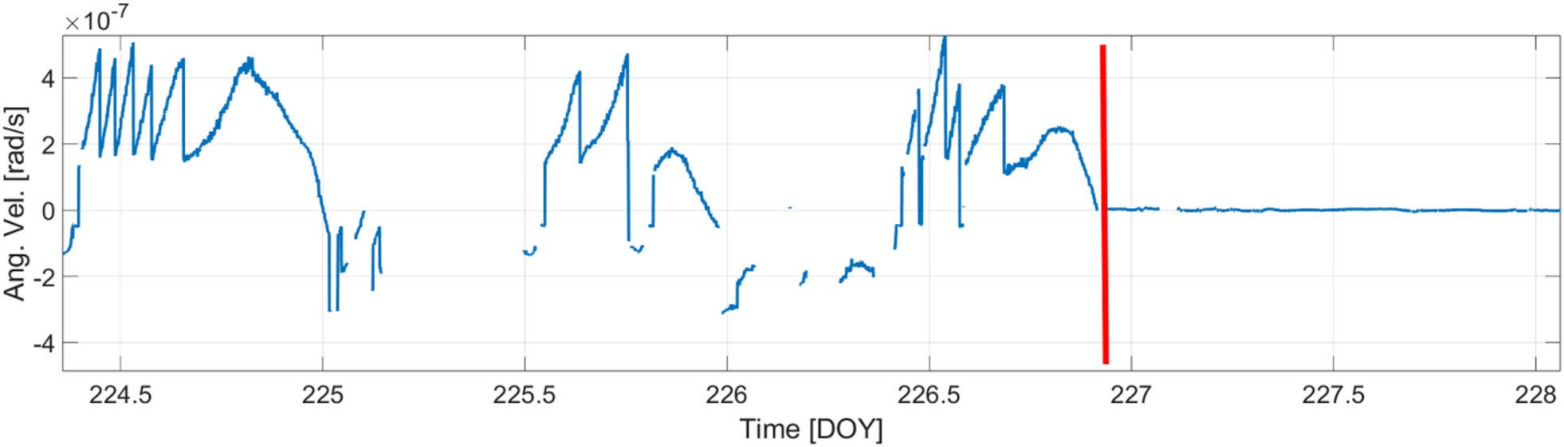
*ω*_*s*_ from August 12 to 15, 2023; the X-axis represents the day of the year (DOY); the red vertical line indicates the occurrence of the *bang* at 22:00 UTC on August 14 (DOY: 226).

The final outcome of the selection is summarized in Figure 17, where the ratio *ρ* of the selected good data points over the total points are plotted on a daily basis. It is possible to observe how the ratio is small and affected by strong fluctuations in the DOY 130–190 interval. Overall, around 65% of data are kept in the analysis after the selection. Typically *ρ* > 90% for *GINGERINO*, our analysis indicates that such a low ρ value can be attributed to the fact that the apparatus was frequently affected by external forces, most frequently for about 55 days after May 11, but less affected after the occurrence of the bang. The 55 days period corresponds to the rapid increase of the curve related to the *MP4* station shown in Figure 6B. Moreover, Figure 16 shows that the fringe contrast *C* around May 11 and at the time of the bang the contrast has abruptly decreased, and the tiltmeter co-located with *GINGERINO* shows a few mrad change of inclination in the West-East direction, see Figure 8. Figure 18 shows *ω*_*s*_ around August 14 (DOY: 226).

**Fig. 19:**
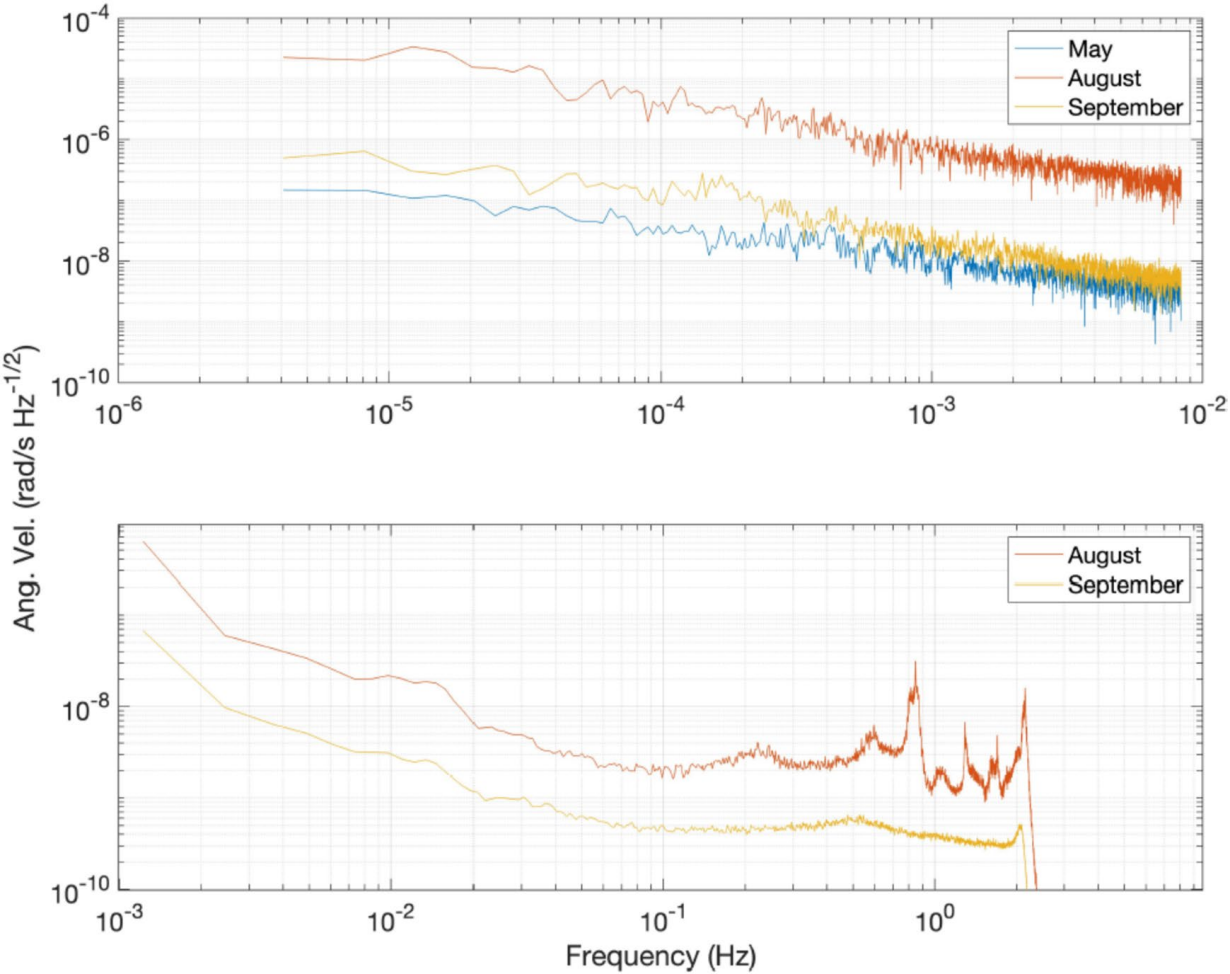
Comparison of *ASD* of *ω*_*s*_ in data portions corresponding to the beginning of May and to September 2023, and a dataset corresponding to the period between July 15 (DOY: 196) and August 15 (DOY: 227). The bottom panel reports the *ASD* of two selected data sets of *ω*_*s0*_ having a duration of about 15 hours each, corresponding to data acquired between July 15 and August 15, and in September.

Finally, in an attempt to further analyze *GINGERINO* behavior in the period of interest, we analyze the Amplitude Spectral Densities (*ASDs*) of *ω*_*s*_ evaluated for three distinct periods, at the beginning of May, between July 15 (DOY: 196) and August 15 (DOY: 227), and in September, 2023. To investigate the low-frequency components, we selected 7 days for each of the three groups. In these three datasets, bad points were replaced with zeroes. To examine the frequency region around 1 Hz in more detail, two different sets were selected from the second and third groups. These corresponding datasets contain only good data and have a length of approximately 15 hours. The resulting *ASD*s are reported in Figure 19. The obtained ASDs suggest that, at the time of the *bang* event (August data), the overall level of instrumental noise increased, accompanied by a rise in the signal at specific frequency bands, around and slightly above 1 Hz.

### Seismological analysis, more measurements and comparison

**Fig. 20:**
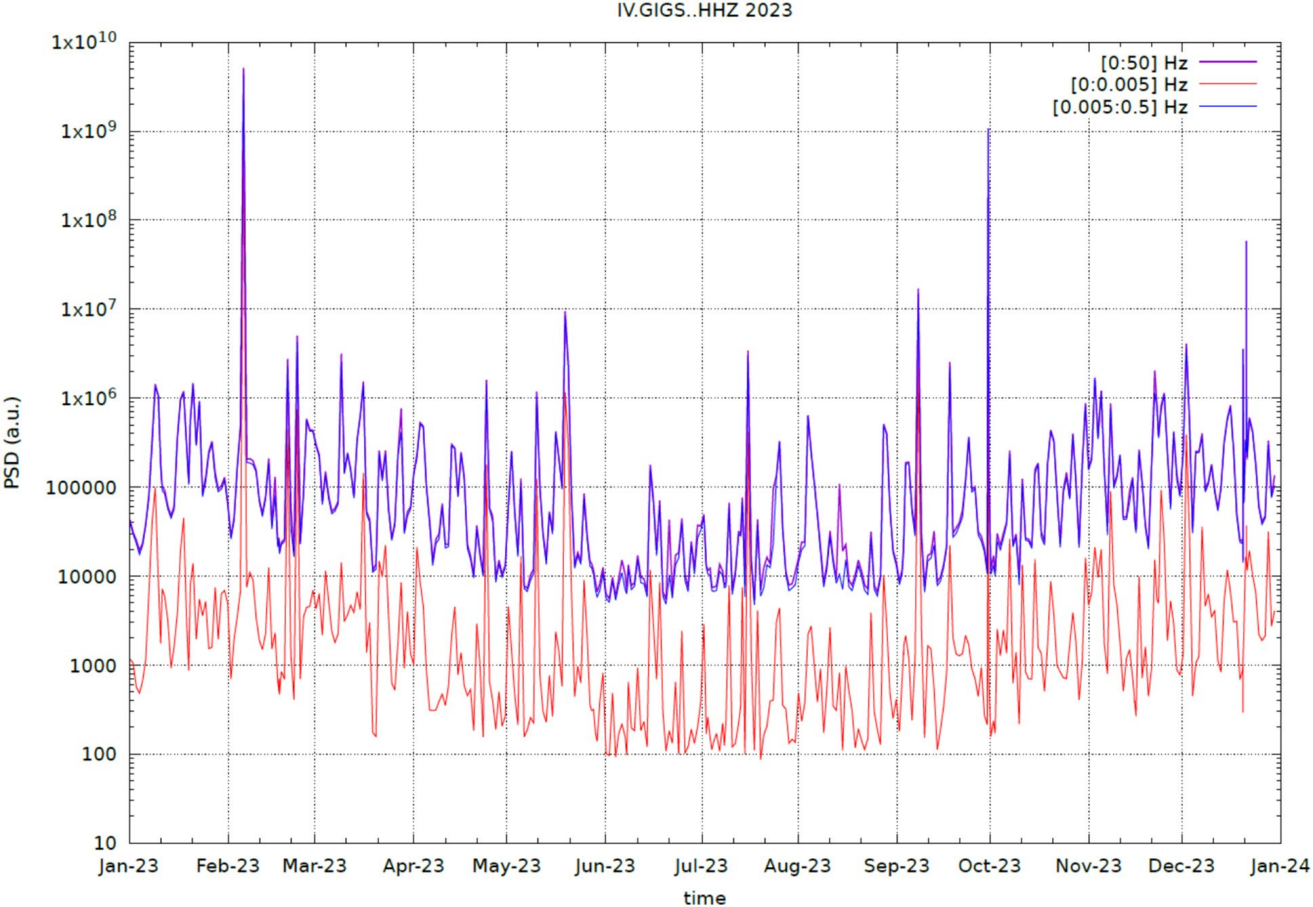
*GIGS* (vertical component) *PSD* total values in arbitrary units (a.u.) throughout 2023 as function of time band passed in different frequency bands (see legend). There is no evidence of particular anomalies in the period May-August 2023. The decrease of *PSD* amplitude in the period June-September is linked to marine noise, which is less significant in the summer periods. The *PSD* is computed over 6-hour time windows overlapped at 50% as in the Welch method^64^.

The analysis of data from the *INGV* seismic station *IV.GIGS* was also extended to a longer time scale (https://terremoti.ingv.it/en/instruments/station/GIGS). Unlike *GINGERINO*, *GIGS* is not sensitive to the long term variations < 4 mHz (the broadband seismic sensor is a 240 s) and disturbances which affect the *RLG* from May to August 14 (DOY: 226), 2023. Figures 20 shows the *GIGS HHZ* (vertical component) *PSD* total values in arbitrary units (a.u.) throughout 2023 as function of time band passed in different frequency bands (see legend in the figure). The *PSD* is computed over 6-hour time windows overlapped at 50% as in the Welch method^64^: There is no evidence of particular anomalies in the period May-August 2023. The decrease of *PSD* amplitude in the period June-September is linked to marine noise that is less significant in the summer periods. It is also worth noting that the graph of the total window, 0–50 Hz (purple line), is almost identical to that in the 0.005–0.5.005.5 Hz window (blue line), while the total *PSD* at low frequencies (0–0.005.005 Hz, > 200 s as period, orange line) has amplitudes with about 3 orders of magnitude less.

**Fig. 21:**
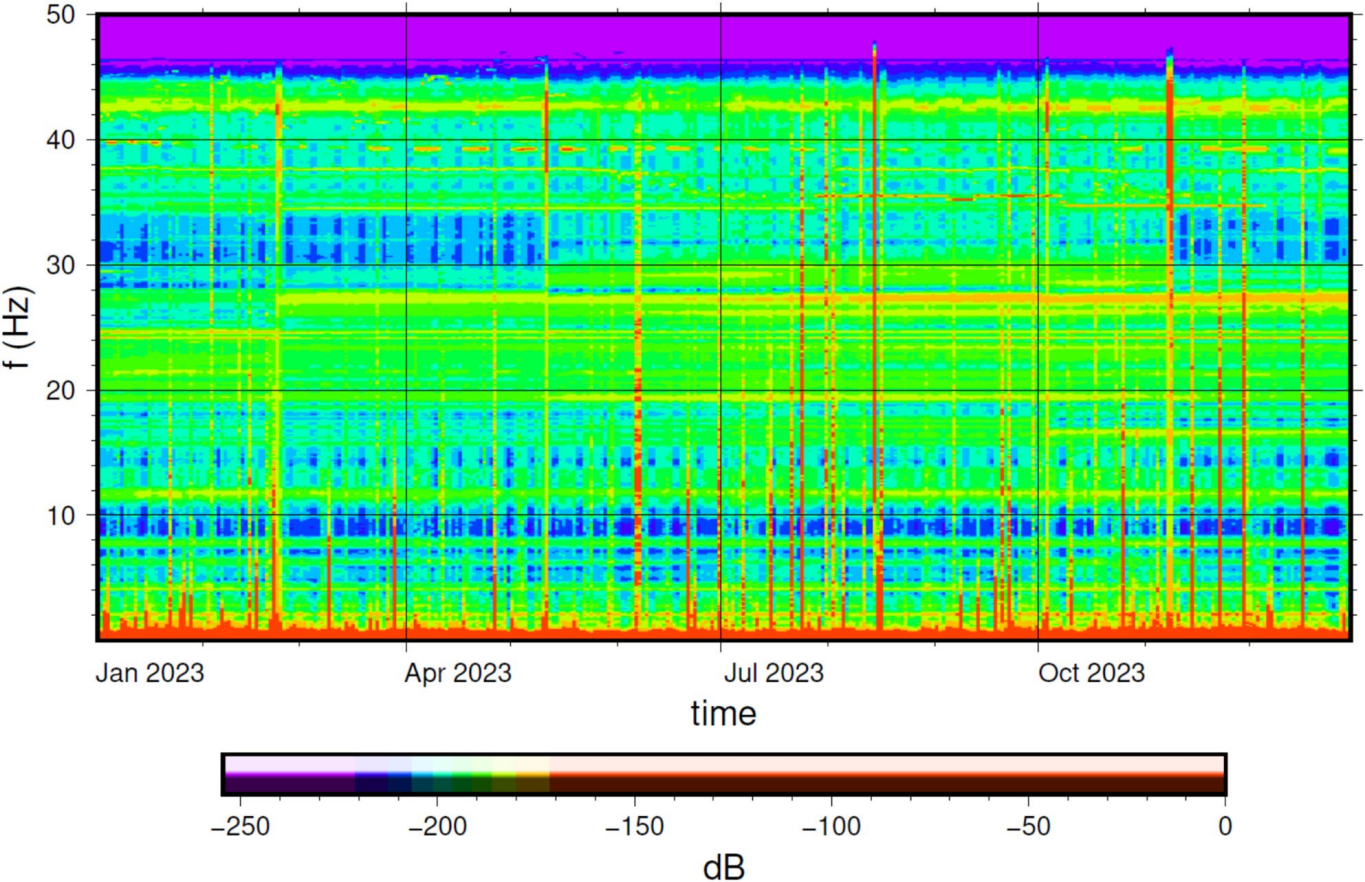
*GIGS* time-frequency analysis (spectrogram) throughout 2023 (vertical component). The seismic data is corrected for the sensor response and is shown in *dB* relative to maximum. The *PSD* is computed over 6-hour time windows overlapped at 50% as in the Welch method^64^. A few significant peaks are visible above 40 Hz, corresponding to the mountain *bang* event of August 14, personnel interventions near the *GINGERINO* sensor (*GIGS* station is co-located, see Figure 9) and probably other similar *bang* events with minor amplitude.

Figures 21 show the *GIGS HHZ* (vertical component) spectrogram throughout 2023 computed over 6-hours time windows overlapped at 50% as in the Welch method^64^,the seismic data is corrected for the sensor response and shown in dB relative to maximum. Most of the energy recorded is contained in the very low frequency band (below 1 Hz). In addition to the obvious *bang* event of August 24, occasionally, the very high frequencies peaks (up to 40 Hz) are usually correlated to high anthropic noise produced in the experiment halls of *LNGS-INFN* and also to the personnel interventions near the *GINGERINO* sensor (*GIGS* station is co-located, see Figure 7, *bottom*).

**Fig. 22:**
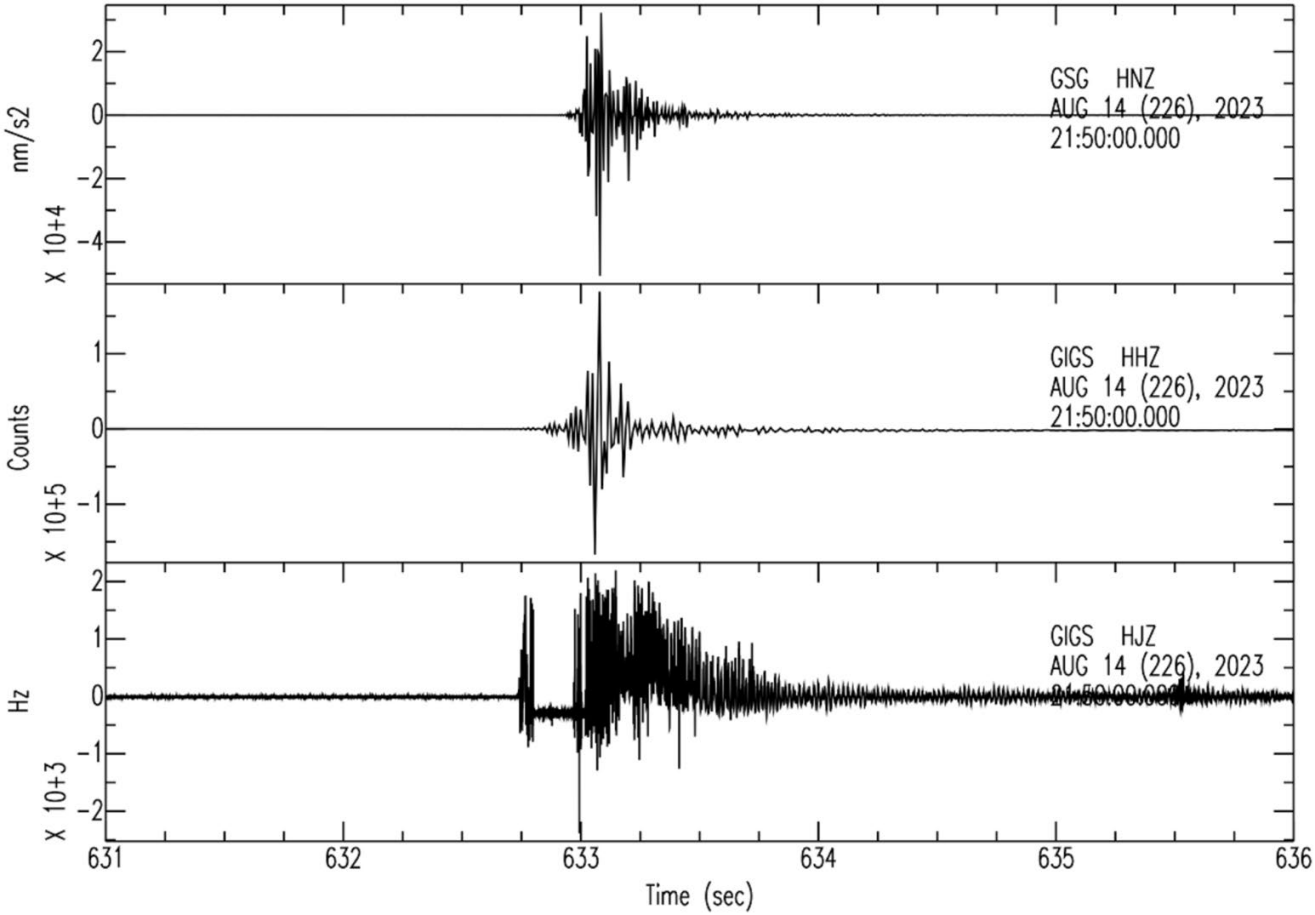
Five seconds of vertical seismic traces compared to rotational *GINGERINO* signal, amplitude is expressed in nm/s^2^ for GSG, in counts for GIGS and in Hz for *GINGERINO*. From top to bottom: *GSG* (*HNZ*, vertical component), *GIGS* (*HHZ*, vertical component), and the rotational signal recorded by *GINGERINO* ring laser (*HJZ* component). Time is in seconds, starting from 21:50 *UTC* on 14 August (DOY: 226).

**Fig. 23:**
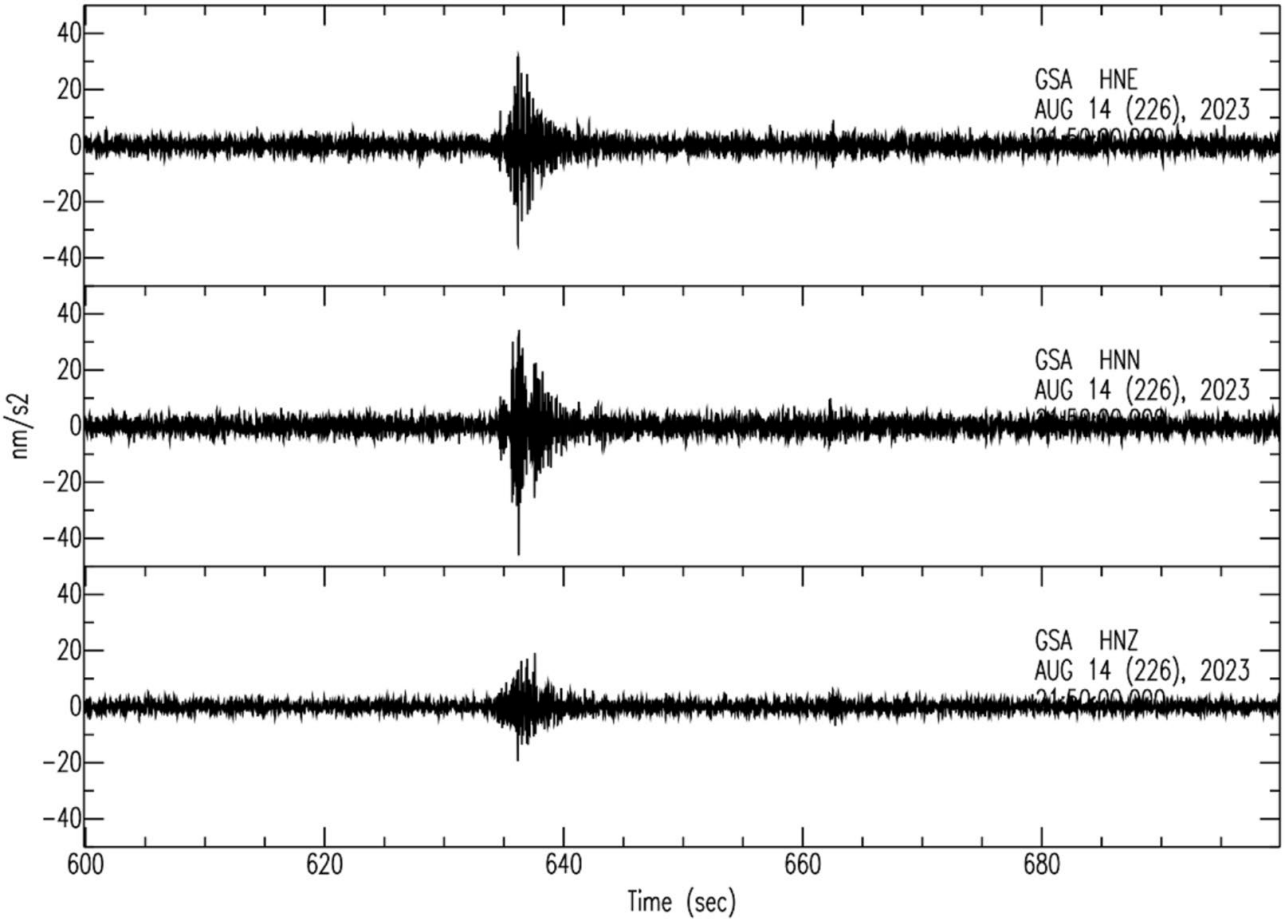
Accelerometer traces by *GSA*, (https://ran.protezionecivile.it/IT/stazioni.php); the amplitude is in nm/s^2^; time is in seconds, starting from 21:50 *UTC* on 14 August (DOY: 226). *HNZ*, *HNN* and *HNE* are the Vertical, North-South and East-West components respectively; the waveforms were filtered in the 0.5–15.5 Hz frequency band.

At *LNGS-INFN*, there are also accelerometer stations, both underground and on the surface, *GSG* and *GSA* respectively; these are part of the *RAN* (*Rete Accelerometrica Nazionale* – https://ran.protezionecivile.it/IT/quakelive.php). The *GSG* is located at about 150 m distance from *GINGERINO* and *GIGS* sensors (*GSG* in Figure 4) while *GSA* is located near the *LNGS* external offices (*GSA* in Figure 3). The very few seconds of the mountain *bang* event, as seen from the vertical components of the seismometer *GIGS*, the accelerometer *GSG* and from *GINGERINO* are compared (see Figure 22). It is conservative to remember that *GINGERINO* was working in very disturbed conditions, in fact for a fraction of second the laser turned off, as already shown previously and as visible in this figure. Data recorded are similar in shape and duration of the *bang* event; the difference in frequency content is related both to the sampling frequency settings of digitizers (200 *sps* for *GSG* and 100 *sps* for *GIGS*) and, above all, for the improved low-frequency response of the *GIGS* broadband sensor (Trillium 240 s). What is visible is a common small-amplitude first arrival before the maximum amplitude phase arrival, which looks higher for the *EW*-component of *IV.GIGS* station and for the *NS*-component of *IT.GSG* station. This suggests a signal polarization difference probably related to the location of the source or perhaps due to an imperfect orientation of the horizontal components. At first glance, this shape of the data would suggest the presence of P wave’s arrival (common small-amplitude) and subsequently S waves generated by a nearby seismic source. The difference in times is approximately 0.35 s that corresponds to a distance of approximately 2.7 km. Since a loud *bang* event was heard, it is very likely that the two arrivals can be identified as compressional waves generated by the *bang* travelling in the rock and immediately after the acoustic signal, propagated in the air that impacts on the seismic sensors. In this case, using the speed of sound in air, 0.343 km/s and the speed of compressional waves in the limestones of the central Apennines, 3.8–5.0.8.0 km/s (Montone and Mariucci^65^ and references inside), we obtain a distance from the source of about 130 m. Since the same arrival times were found at *GIGS* and *GSG*, it is presumable that the origin of the large *bang* event comes either from the north-east part of the underground laboratories or from the opposite part. The *GSA* accelerometer station is located outside the underground laboratories (see Figure 3) more than 6 km away from *GIGS* seismic station. In this case, some energetic wave is barely perceptible about 1.5 s after the other two stations compatible with a compressional wave velocity of 4 km/s. Figure 23 shows the *GSA* 3-component waveforms, filtered in the 0.5–15 Hz frequency band.

**Fig. 24:**
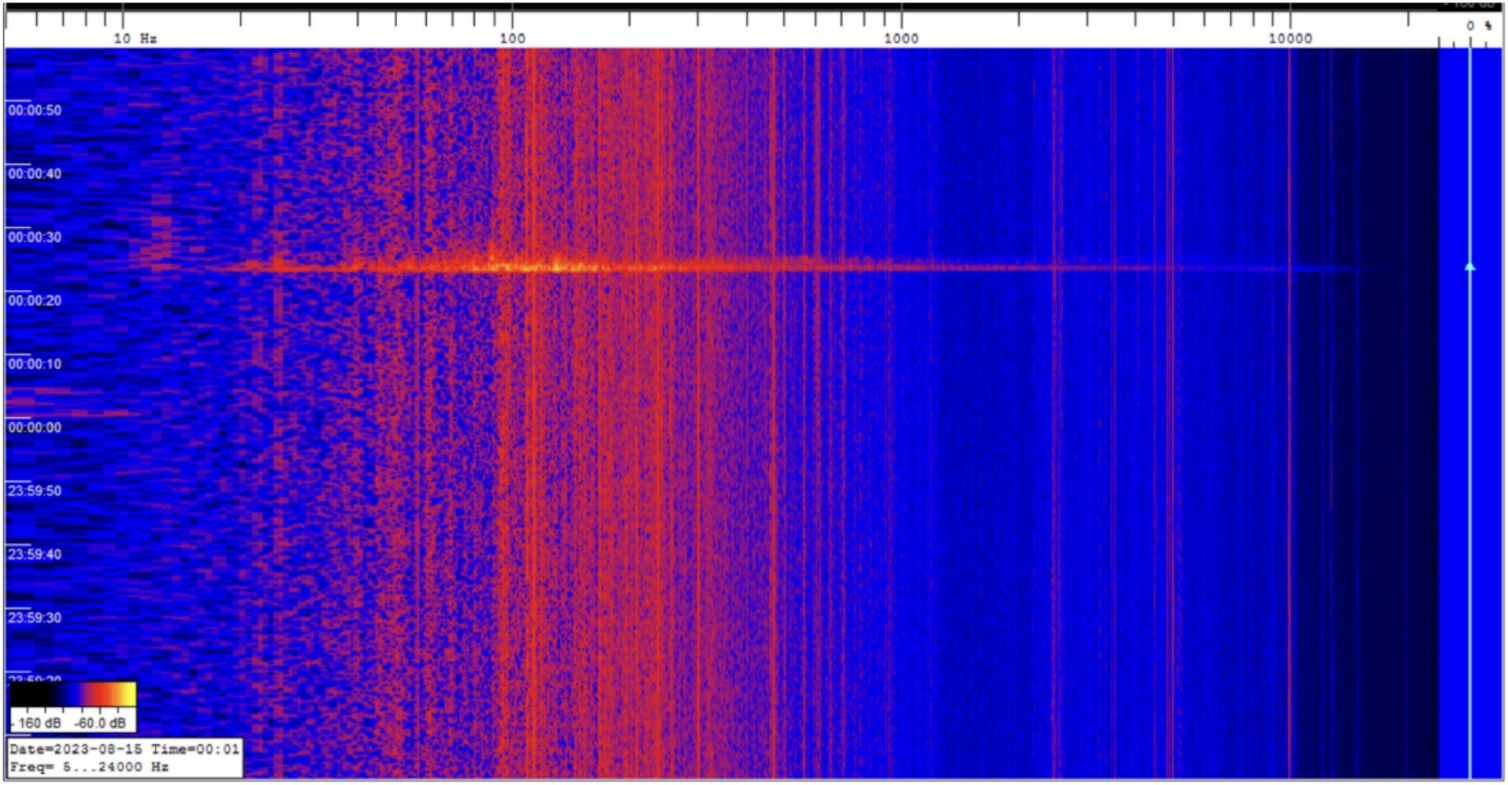
Acoustic signal as recorded during the mountain *bang* event, local time (*UTC*+2 hours); see Figure 4 for position of acoustic sensor.

Inside the underground laboratories (*LNGS-INFN*), there is also a microphone for acoustic measurements (see Figure 4). The *bang* event was recorded between ∼20 and ∼30 seconds after 00:00 local time on 15 August 2023 (corresponding to 22:00 UTC on 14 August 2023), more or less in agreement with the timing of the *GIGS* and *GINGERINO* detections, within the precision of the instrument. In Figure 24, the acoustic detection of the boom is shown; the frequencies involved range approximately from 20 to 15000 Hz, with maximum intensities around 70–200 Hz.

**Fig. 25:**
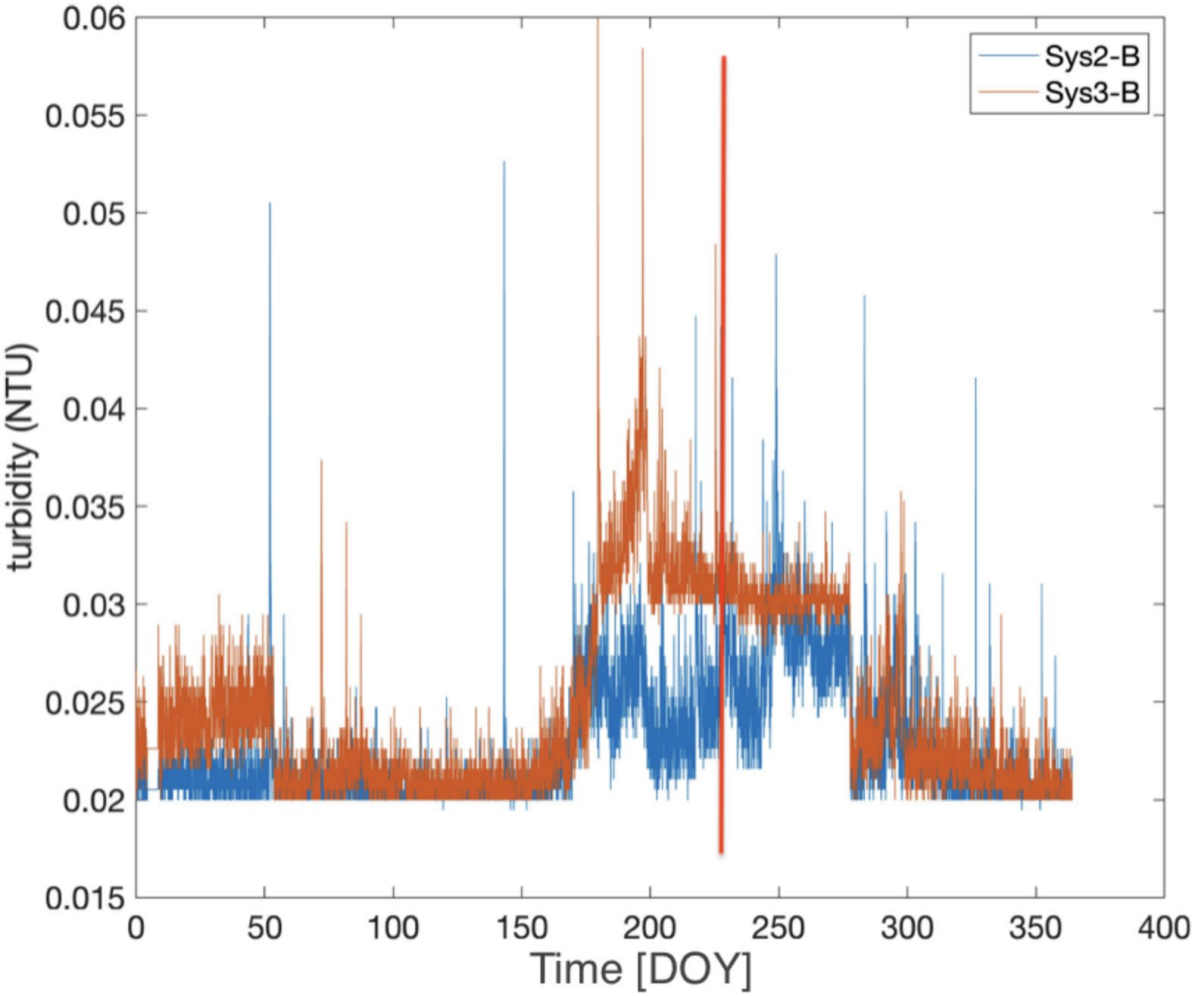
Turbidity at *MP3* site (see Figures 1 and 3), provided by water supply company (https://www.ruzzo.it/) and measured in *NTU*. The two sensors are situated in the right highway barrier L’Aquila-Teramo direction (*Sys2-B*) and in the left highway barrier Teramo-L’Aquila direction (*Sys3-B*) respectively. Turbidity presents higher values between the second half of June and the first half of October. Please, note that the turbidity signals is about 120 days large and shifted with respect to *MP1* and *GINGERINO*. The red vertical line indicates the occurrence of the *bang* at 22:00 UTC on August 14 (DOY: 226).

In the northern drainage area of Gran Sasso motorway tunnel (*MP3* site, see Figures 1 and 3) there is a water supply company (https://www.ruzzo.it/) from which data on the turbidity and water flow rate were requested. The Figure 25 shows turbidity data in *MP3* site: the two sensors are situated respectively in the right highway barrier L’Aquila-Teramo direction (*Sys2-B*) and in the left highway barrier Teramo-L’Aquila direction (*Sys3-B*). Turbidity is measured in *NTU* (*Nephelometric Turbidity Unit*) and it means that the instrument is measuring diffused light from a sample at an angle of 90 degrees with respect to the impinging light. Turbidity presents higher values between the second half of June and the first half of October 2023, compatible with the anomalies recorded by *GINGERINO* (see Figures 15, 16, 17 and 18).

**Fig. 26:**
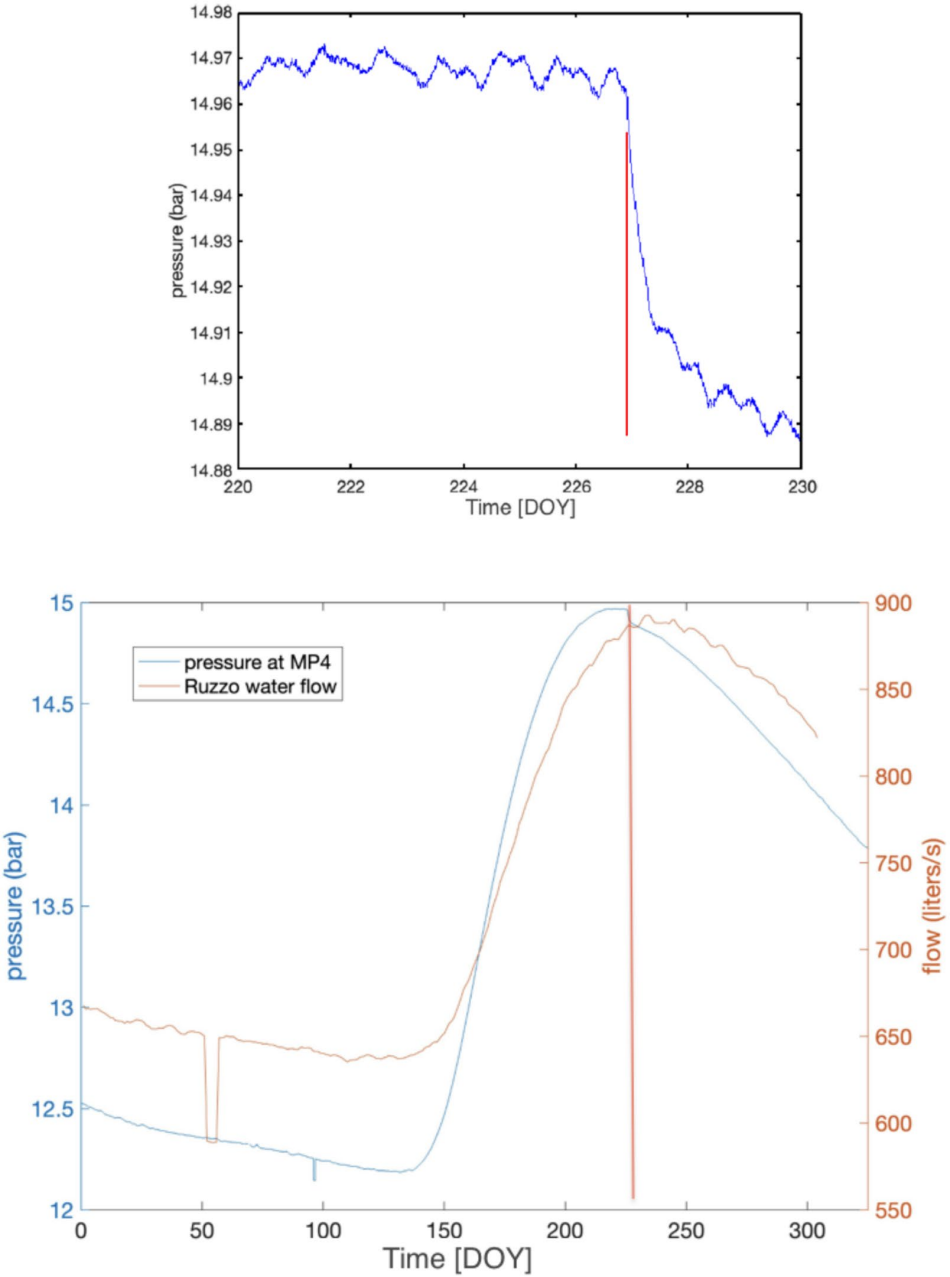
Top: Detail of the hydraulic pressure variation at *MP4* site (see Figures 1, 3 and 4), zoomed at the time of the *bang* event, from 220 (August 8) to 230 (August 18) day of 2023 year. The beginning of the rapid loss of pressure corresponds exactly to the time of origin of the *bang* event. Bottom: Water flow rate at site *MP3* compared to the hydraulic pressure at site *MP4* for the entire year 2023; after the *bang* event of August 24 (DOY: 236) it is possible to notice, a few days later, a slight increase in the flow rate coming out of the tunnels (site *MP3*). The red vertical line indicates the occurrence of the *bang* at 22:00 UTC on August 14 (DOY: 226).

As previously stated, groundwater hydraulic pressure was measured at monitoring point *MP4* (see Figures 1, 3 and 4 for position) with high sampling (20 Hz), and yearly variations were shown in Figure 26 (*top*). Here we point out, after describing other instruments detections, that there was a sudden decrease of hydraulic pressure measured at *MP4*, are about 70 mbar that corresponds to 70 cm of equivalent height lost in approximately 24 hours (see Figure 26, *top*). The beginning of the rapid loss of pressure corresponds exactly to the time of origin of the *bang* event. The Figure 26 (*bottom*) shows both the water flow rate at site *MP3* compared to the hydraulic pressure at site *MP4* for the entire year 2023; after the *bang* event of August 14, it is possible to notice, a few days later, a slight increase in the flow rate coming out of the tunnels (site *MP3*).

## Discussion and conclusion

The present analysis reports observations from multi-parametric instruments, installed inside and outside the Gran Sasso aquifer (Central Apennines, Italy), mainly from May to August 2023, culminating in a large *bang* event observed through the same sets of experimental data. For the first time, a highly sensitive *RLG*, *GINGERINO*, has been added to the analysis.

The hydrometric measures in the *MP2* and *MP3* sites agree both with hydraulic pressure measurements at *MP4* in the underground site and with data from the *Ring Laser Gyroscope GINGERINO.* The hydrometric data showed by an initial period of invariability, which ends with the onset of a sudden and sustained increase from May 2023 to August 2023 for intense rainfall in the spring season. The culminating *bang* event finds evidence and a good temporal coincidence in hydrometric level and hydraulic pressure measurements. The *IV.GIGS* seismometer, *IT.GSG* accelerometer, acoustic sensor and *GINGERINO* also are in agreement with the *bang* event observation, as for timing and common frequencies. An important part of the signal seems to be acoustic, as reported both by the *LNGS-INFN* guards, and as registered by the *INGV* microphone (see Figure 24). In hydrogeology, these observations are often linked to the breaching of permeability boundaries or to turbulent water flows, as demonstrated by the multi-parametric analysis. *GINGERINO* provides a very strong *bang* signal. It is important to remark that *GINGERINO* is one of the first *RLG* prototypes based on an optical cavity done connecting different pieces and equipped with mechanical tools to align the mirrors cavity. One of the main problem of this kind of *RLG* is that occurrence of external forces can misalign the cavity and the read-out optical circuits, in this conditions instrumental rotation can occur, and, acoustic signal can affect the measurement, for these reasons very likely most of the recorded large signal is of instrumental origin. On a longer time scale observation, the analysis demonstrates that *GINGERINO* has been continuously perturbed from May 10 to August 14, in good time coincidence with hydro-geological data, in a good coincidence with turbidity measurements of *MP3* site. The apparatus was not working in optimal conditions, but the quality of the interference was good enough to make a suitable investigation. Despite the technical problems, *GINGERINO* continued to function recording three local earthquakes in a very good resolution (see Figures 9, 12 and 14).

The available *GINGERINO* data between May 1 and September 24, 2023, have been analyzed and they indicate that, around day of year (DOY) 130, corresponding to May 10, an unexpected change is recorded, affecting the general alignment of the *RLG*. Unfortunately between days 130 and 131 (May 10 and 11) most data are missing and details are not available. The only evidence is the large degradation of fringe contrast, unusual in a time interval of 1 day, certainly due to misalignment caused by external forces larger than typical conditions. Moreover, there is clear continuous evidence of deterioration of the alignment of the cavity and of the readout system up to the *bang* event. Since May up to August 14, the angular rotation measured by *GINGERINO* is of the order of fractions of μrad/s, with 1 minute bandwidth, more than 50 times larger than typical conditions. This large motion can be due to instrumental cavity rotation induced by the frequent external perturbations. The standard analysis routines to remove laser disturbances have been successfully applied, mitigating the laser non-linearity, but without reducing the signal amplitude. After the *bang* event, the fringe contrast further deteriorated, *GINGERINO* remained operative in more stable conditions.

The seismic observations lead to localize the *bang* event at a distance of about 130 m from the seismic station *IV.GIGS* and from the accelerometric station *IT.GSG*. Since the same arrival times were recorded, we can argue that the origin of the *bang* event comes either from the northeast part of the underground laboratories or from the opposite part.

Finally, the sudden drop in hydraulic pressure at the *MP4* site (70 mbar in about 24 h) which occurred immediately after the *bang* event suggests a local event involving both rocks and groundwater flow within the Gran Sasso aquifer, which modified the flow-path of groundwater near the underground laboratories. The most probable explanations, alternatively or in combination: i) a breaching of a hydraulic barrier by a fracture movement; ii) the reactivation of a karst conduit by groundwater flow excess.

In summary, the present work focuses on long-term observation from multi-parametric environmental monitoring instruments concentrated inside and outside the Gran Sasso aquifer (Central Apennines, Italy), mainly from May to August 2023, culminating in a strong significant event (*bang*) observed through the same sets of experimental data: *GINGERINO Ring Laser Gyroscope* (*RLG*), seismometers, accelerometers, tiltmeters, hydraulic pressure and acoustic sensors.

This is the first time that a highly sensitive *RLG* has been used together with hydraulic observations inside a large massif aquifer. The multi-parametric observation of the Gran Sasso massif provides an unprecedented insight on the inner dynamics of the mountain. The Gran Sasso aquifer showed a behavior, from May to August 2023, different from the previous years, especially in terms of the hydrometric level and water pressure measured in two relevant monitoring points. Observations of the *GINGERINO* data in such periods show a particularly perturbed signal, since May 10 up to the occurrence of the bang, giving hints of water and possibly slow rock movements. The water turbidity also shows an anomaly lasting for the whole period of May-August 2023. It is important to remark that the turbidity signals is delayed by roughly twenty days with respect to *MP1*, while the anomaly of the *GINGERINO* data are in good temporal coincidence with *MP1*, providing a clear demonstration that the *bang* was connected to the aquifer dynamic. Such perturbed period ended with a strong *bang* event, detected by the *RLG*, the seismometers, accelerometers, and by the aquifer monitoring. The sound of the *bang* was also clearly audible to the guards and recorded by an acoustic sensor. The consequences of this event for groundwater flow in the aquifer and in the drainage system of the highway tunnels are still unknown, and it is strongly suggested to continue to look into these phenomena. Considering the large amount and diverse origin of the data, it can be concluded that such events are not very frequent, but that further similar conditions are not so unlikely. At the same time, this is a clear demonstration of the active hydrodynamics of the Gran Sasso aquifer, where the combination of active tectonics also at the local scale, with an on-going karst evolution, express the not completely steady state of the groundwater flow, in a system perturbed also by the highway tunnel drainage.

The present study opens to a new multi-parametric approach, in investigating the mountain aquifer inner hydrodynamics, with the help of the *RLG* technology, in rotational seismology, which achieves notable sensitivity and, above all, at frequencies very close to 0 Hz.

## Data Availability

*GINGERINO *datasets generated and/or analyzed during the current study are not publicly available, since *GINGERINO*  is an apparatus still under development, but are available from the corresponding author on reasonable request. Seismic data from the *GIGS* station are public and can be downloaded at [https://eida.ingv.it/en/] Accelerometer data from *GSA* and *GSG *stations are public and can be downloaded at [https://ran.protezionecivile.it/IT/quakelive.php] Hydrometric and acoustic data shown and/or analyzed during the current study are not publicly available but are available from the corresponding author on reasonable request.
